# The role and therapeutic potential of glucose metabolism in multidrug resistance of cancer

**DOI:** 10.3389/fcell.2025.1584630

**Published:** 2025-06-19

**Authors:** Qijing Wang, Kai Li, Liang Li, Qin Li, Yanyu Qi, Kai Liu, Hang Yuan, Ping Lin

**Affiliations:** ^1^ Division of Abdominal Tumor Multimodality Treatment, Cancer Center and Lab of Experimental Oncology, State Key Laboratory of Biotherapy, West China Hospital, Sichuan University, Chengdu, Sichuan, China; ^2^ Department of Oncology, The Third People’s Hospital of Chengdu, Affiliated Hospital of Southwest Jiaotong University, Chengdu, Sichuan, China; ^3^ Chengdu Qingshan Likang Pharmaceutical Co., Ltd., Chengdu, Sichuan, China

**Keywords:** multidrug resistance, glucose metabolism, cancer metabolism, glycolysis, cancer therapy

## Abstract

Cancer represents a serious threat to human health and life. Despite recent advances in the cancer therapy that significantly extend patient survival, many individuals still undergo drug resistance, even to multiple chemotherapeutic drugs, known as multidrug resistance (MDR). MDR causes the treatment failure and promotes the risk of tumor recurrence and metastasis, which has been a critical clinical challenge. The molecular mechanisms for cancer cells developing MDR are complex and largely unclarified. ATP-binding cassette (ABC) transporters-mediated enhanced drug efflux and glucose metabolic reprogramming have been recently identified as key factors that limit drug efficacy. In addition to regulating glucose metabolism, several glycolytic enzymes exhibit aberrant cellular localization, including translocation to the nucleus, cell membrane or mitochondria, which imparts their non-classical pro-oncogenic functions to facilitate tumor progression and MDR. In this review, we summarize the roles and molecular insights of glycometabolic enzymes in MDR progression and discuss existing therapeutic strategies of targeting glucose metabolic enzymes for overcoming MDR.

## 1 Introduction

Malignant tumors are a serious threat to human health and are a significant global public health concern, with 19.29 million new cancer cases diagnosed and 9.96 million cancer-related deaths occurring worldwide in 2020. Surgical intervention may be suitable for select patients, but for most individuals with malignant tumors, treatment includes various modalities, such as radiotherapy, chemotherapy, immunotherapy, endocrine therapy, gene therapy, and targeted therapy. Among these modalities, chemotherapy is still one of the most effective methods for tumor treatment. However, the main factor underlying the clinical failure of chemotherapy, especially in recurrent and metastatic tumors, is the acquisition of chemoresistance. A recent study has shown that more than 90% of cancer-related deaths are attributed to drug resistance (Bukowski et al., 2020). Despite tremendous advances in tumor biology and treatment, including the development of immunotherapies and targeted therapies with significant antitumor activity, patients still exhibit resistance to clinical treatment (Kang et al., 2007; Vasan et al., 2019; Boumahdi and de Sauvage, 2020).

Multidrug resistance (MDR) refers to the cross-tolerance of tumor cells to multiple chemotherapy drugs that are structurally and functionally unrelated (Cao et al., 2019), with the sensitivity of a tumor to different drugs decreasing or even disappearing after repeated exposure to one drug. Tumor drug resistance can be classified as primary or acquired resistance (Gide et al., 2018). Primary drug resistance refers to the intrinsic resistance of tumor cells to a particular anticancer drug, regardless of exposure to the drug. Acquired drug resistance is defined that patients have a good initial response to an anticancer drug followed eventually by clinical progression of disease. Multiple mechanisms can lead to the development of drug resistance in tumor cells, and these mechanisms can be broadly classified into two types: drug-related resistance mechanisms and cellular response-related resistance mechanisms. Drug-related resistance mechanisms include increased drug efflux via ABC transporters, decreased drug absorption caused by changes in lipid metabolism or drug carrier family member expression, and altered drug targeting. Tumor cells can protect against drug toxicity in response to exposure by increasing DNA damage repair activity; modulating cell death mechanisms, metabolic reprogramming, and microRNA expression; and altering the tumor microenvironment (TME) and epigenetics. The mechanisms of drug resistance are complex, which generally results from the interactions and influence of multiple drug resistance-related factors. Metabolic disruption is considered one of the most important causes of drug resistance (Lv et al., 2022). To protect against drug toxicity, tumor cells usually alter metabolic pathways, including the glycolytic pathway, glutamine metabolism pathway, serine synthesis pathway, oxidative phosphorylation (OXPHOS) pathway, pentose phosphate pathway (PPP), fatty acid oxidation pathway, and methionine metabolism pathway. Metabolic reprogramming is also a hallmark of tumor cells (Konieczkowski et al., 2018). The role of reprogramming of glucose metabolism in tumor drug resistance has been a major research focus. Glucose metabolism not only provides the energy and intermediates required for tumor growth and other biological activities, but also produces the crucial reducing power nicotinamide adenine dinucleotide phosphate (NADPH) for anabolic reactions and redox balance. Accumulating evidence has revealed that targeting glucose metabolism has been widely considered as an effective and promising anticancer strategy. In this review, we summarize the current understanding of glucose metabolism (including glycolysis, OXPHOS, PPP pathway and corresponding metabolites) on MDR, and discuss the potential and available targets for preventing, delaying or reversing drug resistance.

## 2 The potential mechanism of MDR

### 2.1 Adenosine triphosphate (ATP)-binding cassettes (ABCs) protein family

The mechanisms underlying the development of chemotherapy-induced MDR are associated with multiple factors, including genetic and epigenetic alterations, apoptosis resistance, the TME remodeling, and increases in drug efflux and the DNA repair capacity (Bukowski et al., 2020). Notably, the uptake and efflux of antitumor drugs significantly impact the development of MDR in tumor cells, which can result in the presence of only a narrow therapeutic window for most anticancer drugs during chemotherapy. While the delivery of higher doses of therapeutic drugs has been largely successful in cancer treatment, it has failed to prevent the development of drug resistance. To achieve therapeutic benefit, it is necessary to determine the concentration at which the drug remains active with minimal toxicity and then to transport the drug to the appropriate target site. Early studies suggested that transport proteins on the plasma membrane of tumor cells are able to reduce drug uptake and increase drug efflux, leading to a decrease in the intracellular drug concentration and subsequent chemotherapy failure. Elevated expression and activity of these transport proteins is one of the main mechanisms underlying drug resistance. Interestingly, the upregulation of a single transport protein associated with drug efflux is connected to the progression of MDR. Drug resistant variants that arise in cancer cells as a result of prolonged sustained exposure to a single cytotoxic agent also mediate incidental resistance to a broad spectrum of cytotoxic agents. All of these MDR proteins contain multiple transmembrane structural domains (TMDs) and intracellularly localized ATP-binding cassettes (ABCs) belonging to the ABC transporter superfamily. Cancer cells overexpressing members of the widely recognized ABC protein superfamily export various targets and structurally distinct chemicals, leading to MDR in cancer (Roundhill and Burchill, 2012). Currently, at least 17 human ABC transporters have been demonstrated to promote the formation of MDR phenotypes in cancer cells. Among these ABC transporters, P-glycoprotein (P-gp/ABCB1), multidrug resistance-associated protein 1 (MRP1/ABCC1), and breast cancer resistance protein (BCRP/ABCG2) have the strongest ability to induce MDR *in vitro* (Hanssen et al., 2021).

#### 2.1.1 P-gp

P-gp, also known as multidrug resistance protein 1 (MDR1), is an efflux transport protein involved in the absorption, distribution, and elimination of a variety of compounds. P-gp limits drug penetration through the blood‒brain barrier, restricts drug absorption from the intestine into the bloodstream, and promotes hepatobiliary and renal drug clearance (Elmeliegy et al., 2020). Excessive expression of P-gp results in pleiotropic resistance of cancer cells to numerous neutral and ionic hydrophobic antitumor agents, including paclitaxel, doxorubicin and etoposide. Recent studies have shown that although the energy provided by ATP can support the translocation of engaged substrates, the hydrolysis of ATP allows a continuous conformational change that may facilitate the MDR1 binding and associated trafficking of diverse substrates (Robey et al., 2018). P-gp can also counteract apoptosis via the removal of key caspases from cells or inhibition of caspase activity (Teng et al., 2019). Thus, inhibition of P-gp has long been thought to improve the response of tumors to chemotherapy in human patients.

#### 2.1.2 MRP1

MRP1 is an important pharmacological barrier to drug uptake and elimination and has found in most tissues with increased levels in organs. In subcellular organelles such as endocytic vesicles, it may act via an isolator mechanism to prevent drug delivery to intracellular targets (Rajagopal and Simon, 2003). Upregulation of MRP1 has been observed in various cancers, such as acute lymphoblastic leukemia (ALL), breast cancer, acute myeloid leukemia (AML), and non-small cell lung cancer (NSCLC) (Lu et al., 2015). In locally advanced NSCLC, patients with high MRP1 level had a worse histopathologic response to cisplatin-based chemotherapy, and a shorter tumor-free survival and overall survival than those with low MRP1 expression (Sedoris et al., 2010). MRP1 can confer resistance to a family of similar but not identical antineoplastic agents, including anthracyclines, anti-androgens, folate-based antimetabolites, and vinca-alkaloids (Munoz et al., 2007).

#### 2.1.3 ABCG2

Broad substrate specificity ATP-binding cassette transporter G2 (ABCG2), a member of the G subfamily of the ABC protein superfamily, was originally cloned from the anthracycline resistant human breast cancer cell line MCF-7/Adrvp and hence named breast cancer resistance protein (BCRP). ABCG2 is widely expressed and can transport many different compounds, particularly in tissues with barrier and secretory functions. ABCG2 is upregulated in the extracellular vesicle (EV) membranes of neighboring breast cancer cells, and actively pumps chemotherapeutic drugs from the cytosol into the vesicle lumen, resulting in an MDR phenotype (Goler-Baron and Assaraf, 2011).

### 2.2 Regulatory pathways associated with the ABC transport protein family

#### 2.2.1 PI3K/Akt pathway

Phosphatidylinositol 3-kinase (PI3K) is an important lipid kinase involved in intracellular signaling and cascade reactions. Protein kinase B (Akt), a key downstream effector of the PI3K signaling pathway, contributes to regulating various physiological and pathological processes, including apoptosis, proliferation, and metabolism. The PI3K/Akt pathway was found to be activated in multidrug resistant cells and confer the MDR phenotype via integration with upstream and downstream signals as well as enhancement of cancer stem cell characteristics (Liu et al., 2020). Aberrant activation of the PI3K/Akt pathway enables to promote cell cycle progression and survival signals, accompanied by the increase of antiapoptotic gene expression and the reduce of proapoptotic gene expression (Rocha et al., 2014). Apart from upregulation of ABC transporter protein expression, the PI3K/Akt pathway augments the drug efflux ability of ABC transporters by accelerating aerobic glycolysis-mediated the energy supply (Sui et al., 2014; Yamamoto et al., 2014; Wang et al., 2010). Akt may drive TCA cycle flux and lipid biosynthesis by directly phosphorylating and activating ATP-citrate lyase, which protects tumor cells against chemotherapy drugs toxicity (Robey and Hay, 2009).

#### 2.2.2 HIF-1α pathway

Solid tumors are often exposed to a hypoxic environment, in which tumor cells usually exhibit a series of adaptive responses to cope with the unfavorable condition while satisfying the energy and nutrient requirements for their growth and continued proliferation. Hypoxia/hypoxia-inducible factor-1α (HIF-1α) is an oxygen-sensitive subunit of the heterodimeric transcription factor HIF that is inducible expressed under hypoxia. Upregulation of HIF-1α is observed in different type of tumors and HIF-1α activates the transcription of numerous genes, including vascular endothelial growth factor (VEGF), glucose transporters, and glycolytic enzymes whose protein products stimulate angiogenesis for oxygen delivery or accelerate metabolic adaptation to hypoxia. More importantly, HIF-1α not only modulates glucose metabolism to promote the Warburg effect but also regulates the expression of ABC transport proteins. For instance, hypoxia induces extracellular signal-regulated kinase 1/2 (ERK1/2) phosphorylation to activate HIF-1α, which directly binds to the promoter of ABCG2 gene and subsequently enhances ABCG2 transcription, leading to gemcitabine chemoresistance in pancreatic cancer cells (He et al., 2016). Ursolic acid diminishes ABCG2 expression and reverses cisplatin tolerance via blockage of PI3K/Akt pathway mediated activation of HIF-1α in ovarian cancer stem cells (Wang et al., 2016). Additionally, HIF-triggered metabolic reprogramming led to sorafenib resistance in several hepatocellular carcinoma (HCC) cell lines (Bao and Wong, 2021).

#### 2.2.3 c-Myc pathway

C-Myc is a proto-oncogene encoding an HLH-leucine zipper transcription factor that regulates gene expression by binding to an enhancer box sequence. c-Myc is an important mediator of the development of MDR. Overexpression of c-Myc in human mammary epithelial MCF10A cells increased the expression and activity of ABCG2 (Kang et al., 2009), suggesting a role for c-Myc in influencing cellular sensitivity to chemotherapy. Moreover, c-Myc was shown to bind to the ABCG2 promoter when the c-Myc binding site is unmethylated (Porro et al., 2011). In contrast, the inhibition of c-Myc in tamoxifen (TAM) resistant cells decreases cell viability and glucose uptake, and prevents proper regulation of the glutaminase-glutamine synthetase (GLS/GAC-GLUL) system (Miller et al., 2011; Venditti et al., 2002).

**FIGURE 1:**
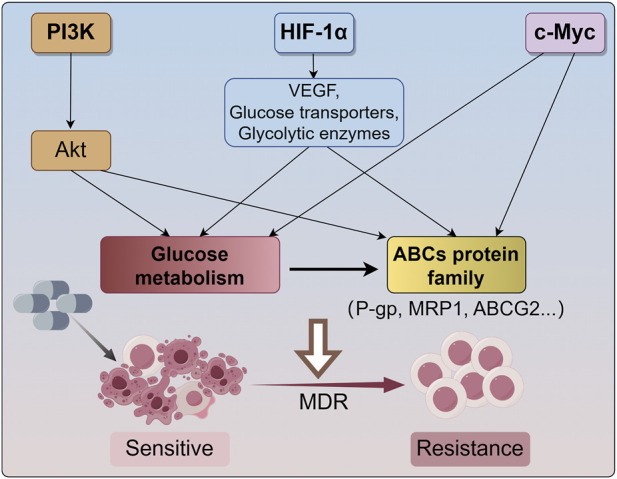
The main mechanism for cancer undergoes MDR. The PI3K/Akt signaling pathway enhances the drug efflux capacity of ABC transporters by facilitating energy supply through aerobic glycolysis. Hypoxia-inducible factor 1-alpha (HIF-1α) activates the transcription of various genes, such as vascular endothelial growth factor (VEGF), glucose transporters, and glycolytic enzymes, thereby modulating glucose metabolism to support the Warburg effect and regulating the expression of ABC transport proteins. Additionally, c-Myc influences glucose metabolism and augments the expression and activity of ABCG2. These signaling pathways reprogram glucose metabolism and impact the expression of ABCs transporter proteins (P-gp, MRP1, ABCG2, etc.), resulting in cancer MDR. MDR, multidrug resistance; HIF-1α, hypoxia-inducible factor 1-alpha; VEGF, vascular endothelial growth factor; ABCG2, ATP-binding cassette transporter G2; P-gp, P-glycoprotein; MRP1, multidrug resistance related protein 1.

MDR is a multifactorial phenomenon, and the expression of ABC transporter proteins in cells with the MDR phenotype is only one factor associated with the pharmacological escape from chemotherapeutic drugs. Because ABC transport proteins require considerable amounts of ATP to maintain their drug efflux activity, tumor metabolism can be modulated by intermittent switching between anaerobic and oxidative metabolism to meet the demand for ATP due to tumor heterogeneity. Accumulating evidence has reported that cellular metabolism undergoes a shift from OXPHOS to glycolysis in response to hypoxic conditions (Vidal et al., 2018). Consequently, the expression of ABC transport proteins is elevated in tumor cells treated with exogenous pyruvate, whereas glycolysis inhibition obtains the opposite result. Moreover, redox status caused by the balance between reactive oxygen species (ROS) generation from mitochondrial electron transport chain (ETC) and free radical scavenger derived from glycolytic products and PPP pathway has been shown to alter ABC transport protein expression (Cort et al., 2016). These observations indicate that glucose metabolism has profound implications for the expression of MDR transport proteins and the acquisition of MDR in tumor cells (Figure 1).

## 3 Glycolysis and MDR

**FIGURE 2:**
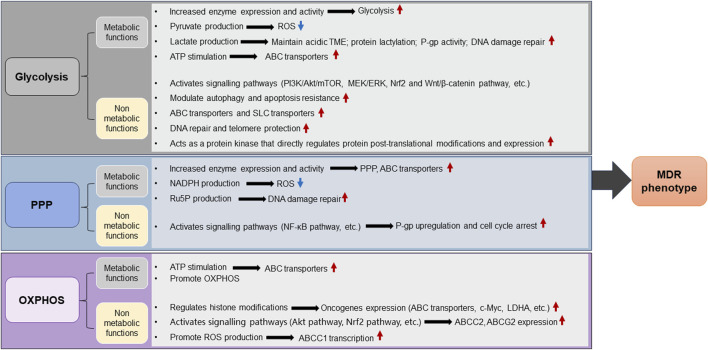
The role of glucose metabolic enzymes and metabolites in promoting MDR of cancer. Glycolysis, OXPHOS, and PPP constitute significant components of glucose metabolism. Tumor cells preferentially utilize glycolysis for energy production even in the presence of a sufficient oxygen supply, a phenomenon known as the Warburg effect. In the glycolytic pathway, tumor cells enhance glycolysis by upregulating the expression levels or enzymatic activity of key metabolic enzymes, as well as promoting the accumulation of metabolites such as lactate and pyruvate, which induces alterations in the intracellular environment and fosters an acidic TME. These metabolic reprogramming events facilitate the scavenging of intracellular ROS or activate lactylation modification-mediated regulatory processes, collectively enhancing tumor resistance to therapeutic agents. Furthermore, glycolytic metabolic enzymes can be aberrantly regulated to perform nonmetabolic functions, facilitating the development of MDR phenotypes in tumor cells by promoting DNA damage repair, autophagy, transporter protein expression, apoptosis resistance and oncogenic signalling pathways. Aberrant expression of metabolic enzymes in the PPP enhances NADPH, Ru5P and ROS levels while accelerating the synthesis of DNA repair precursors or activating signalling pathways, thereby promoting MDR. Upregulation of metabolic enzymes in OXPHOS increases ATP production and accelerates the OXPHOS pathway, thereby enhancing MDR of cancer cells. Additionally, the nonmetabolic functions of OXPHOS can regulate certain proteins expression and activate specific signaling pathways, leading to MDR. Red arrows indicate activation or upregulation, blue arrows indicate inhibition or downregulation. MDR, multidrug resistance; OXPHOS, oxidative phosphorylation; LDHA, lactate dehydrogenase A; PPP, pentose phosphate pathway; TME, tumor mircroenvironment; P-gp, P-glycoprotein; ATP, adenosine triphosphate; ROS, reactive oxygen species; PI3K, phosphatidylinositol 3-kinase; ABC transporters, ATP-binding cassette (ABC) transporters; SLC transporters, Solute carrier transporters; Akt, protein kinase B; mTOR, mammalian target of rapamycin; MEK, mitogen-activated extracellular signal-regulated kinase; ERK, extracellular regulated protein kinases; NADPH, nicotinamide adenine dinucleotide phosphate; Ru5P, ribulose 5-phosphate; Nrf2, nuclearrespiratoty factor 2.

In the presence of a sufficient oxygen supply, the energy required for the growth of normally differentiated cells is provided mainly via OXPHOS, however, tumor cells prefer to use glycolysis to generate energy, a phenomenon also called the Warburg effect or aerobic glycolysis. Although aerobic glycolysis produces much less efficiency of energy than OXPHOS, this process is accompanied by greater reduction of nitrogen and carbon consumption to provide metabolic intermediates to support the rapid growth and proliferation of cancer cells and even to assist drug resistance (Liu et al., 2023). Glycolysis initiates the accumulation of lactic acid and the formation of an acidic and hypoxic microenvironment conducive to MDR (Luo et al., 2016). Highly active aerobic glycolysis is a common hallmark of multidrug resistant cancer cells to fuel their nutrient and survival needs via increase of glucose uptake (Yoo et al., 2019). Glycometabolic adaptation mediated by Akt/mTOR/c-Myc signaling has been confirmed to facilitate the insensitivity of drug resistant leukemia cells to adriamycins (Zhang et al., 2017). Higher rates of lactate production and glucose consumption were observed in doxorubicin resistant breast cancer cells than those in doxorubicin-sensitive cells (Ahmadpour et al., 2021). Disturbing cellular lactate homeostasis through the miRNA-124-mediated lactate transporter 1 suppression improves paclitaxel resistance in breast cancer cells (Hou et al., 2019). Inhibition of glycolysis causes severe depletion of cellular ATP contents in cancer cells with mitochondrial respiration defects and effectively elicits apoptosis in multidrug resistant cells (Xu et al., 2005). Uncontrolled proliferation of cancer cells leads to deprivation of glucose, which acts as a second messenger that triggers distinct stress signaling pathways. In parallel, proliferation-related signaling pathways synergistically regulate the glucose metabolic pathway, activating a suite of metabolic enzymes to prevent apoptosis and maintain cell survival, in turn resulting in the MDR phenotype (Bhattacharya et al., 2016; Lin et al., 2019). Furthermore, abnormalities in saccharides (including glycan structures, glycoproteins and their associated enzymes) are closely associated with MDR phenotype, and this observation provides an alternative intervention strategy targeting MDR in cancer treatment (Wang et al., 2021). Therefore, it is generally accepted that glucose metabolism-related metabolic enzymes have a pivotal function in MDR. Herein, we summarise and discuss current information on the role of glycometabolic enzymes in promoting glucose metabolism and MDR in tumor cells (Figure 2), and conclude the molecular mechanisms by which glycometabolic enzymes promote MDR in tumor cells (Table 1).

**TABLE 1:** The mechanism of glucose metabolic enzymes contributes to MDR of cancer.

Metabolic pathway	Protein	Mechanism	Function	References
Glycolysis	HK	Binds to VDAC protein	Promotion ATP synthesis in mitochondria and accelerates glycolysis	Lincet and Icard (2015)
		Competitive inhibition of the binding of Bcl-2 family proteins to VADC	Inhibition of apoptosis and chemotherapy resistance	Pastorino and Hoek (2008)
		Activated by PI3K/Akt/mTOR signaling pathway	Induction of the MDR phenotype	Min et al. (2013)
		Interacts with mTOR to inhibit mTOR activity	Promotion of autophagy and tamoxifen chemoresistance	Liu et al. (2019)
		Phosphorylated by PIM2	Enhances HK2 protein stability and enzymatic activity, induces autophagy, and mediates paclitaxel resistance	Yang et al. (2018)
		Overexpression promotes ERK phosphorylation	Enhancement of MEK/ERK signaling pathway mediated autophagy, leads to chemoresistance through ATP-dependent and ABC transporter-independent mechanisms	Zhang et al. (2018b)
	PGI	Binds to HER2 and activates HER2 phosphorylation and PI3K/MAPK signaling, leads to Akt activation	Promotes apoptosis	Kho et al. (2013)
	PFK	KAT5 acetylates PFKP, which translocates to the cell membrane to recruit p85α to cause PI3K phosphorylation	Activation of Akt enhances PFK1 activation and tumorigenesis	Lee et al. (2018)
		Akt phosphorylates PFKP. Inhibition of polyubiquitination and degradation of PFK	Promotes aerobic glycolysis, cell proliferation and tumor growth	Lee et al. (2020)
		Regulation of PFKFB3 through Akt/ERCC1 signaling	Promotes DNA repair and apoptosis resistance	Jones et al. (2022)
	TPI	Driven by β-catenin/p53 signaling axis	Acts as a tumor suppressor	Fukushi et al. (2022)
		Expression upwards	Partial reversal of the MDR phenotype in cells	Pekel and Ari (2020)
		Oxidative stress and exposure to chemotherapeutic agents stimulate nuclear translocation of TPIs	Enhancement of cancer cell resistance to chemotherapeutic drugs	Lone et al. (2018)
	GAPDH	Translocates to the nucleus and binds to telomeric DNA	Protection of telomeres from chemotherapy-induced degradation and growth inhibition	Liu et al. (2022)
		Interacts with small GTPase Rheb	Regulation of mTOR signaling, possibly involved in MDR induction	Li et al. (2020)
		Complex with aldolase and phosphofructokinase, makes ion pumps consume ATP directly	Possible regulation of drug efflux	Lee et al. (2009)
	PGK	Enhances CXCR4-mediated ERK phosphorylation	Development of resistance to sorafenib	Campanella et al. (2005)
		High expression promotes epithelial-mesenchymal transition	Reduces cellular sensitivity to erlotinib treatment	Qiu et al. (2024), Chen et al. (2015)
		Phosphorylated by ERK, mitochondrial translocation occurs	Phosphorylates PDHK1, inhibits tricarboxylic acid and ROS production, and promotes lactate production	He et al. (2022)
		O-GlcNAc modification, mitochondrial translocation occurs		Cheng et al. (2020)
		Translocates to the nucleus and interacts with CDC7 by phosphorylating casein kinase 2α protein	Recruits DNA helicase to initiate DNA replication and accelerate tumor development	Li et al. (2018)
		Acetylated by ARD1 and then phosphorylated Beclin1 protein	Induction of autophagy to maintain homeostasis	Nie et al. (2020)
	PGAM	Increases nuclear CtIP protein stability	Supports homologous recombination repair of chemotherapy-induced DNA double-strand breaks	Chen et al. (2023)
		Regulation of the BCL-2 pathway	Resistances to cytotoxicity of the androgen receptor inhibitor enzalutamide	Li et al. (2023)
	ENO	Interacts with CMTM6 protein	Enhancement of cellular drug resistance phenotypes	Mohapatra et al. (2021)
	PKM	Interacts with CD44 protein	Promotes glucose uptake and PPP flux, maintaining cellular redox homeostasis and protecting cancer cells from anti-cancer drugs	Tamada et al. (2012)
		NOX4 induced generation of ROS inhibits p300/CBP-associated factor-dependent acetylation and lysosomal degradation of PKM2	Induces etoposide resistance in the renal cell carcinoma model	Shanmugasundaram et al. (2017)
		Translocates to the nucleus, activates β-cyclin and promotes c-Myc transcription	Promotes MDR phenotypes	Yang et al. (2017), Yang et al. (2012)
		Phosphorylation of histone H3 in the nucleus promotes facilitated c-Myc transcription		Yang et al. (2014)
		Influenced by epigenetic modifications that regulate ABCB1 expression	Involves in drug transport	Wang et al. (2023b)
		Phosphorylated by ATM, increased nuclear accumulation and further phosphorylation of CtIP	Enhances homologous recombination repair and tolerance to DNA-damaging drugs	Sizemore et al. (2018)
		Translocates to mitochondria, interacts with BCL-2 and prevents degradation of BCL-2	Leads to apoptosis	Liang et al. (2017)
		ALDH1A3 interacts with PKM2 to promote lactate production inducing XRCC1 undergoes lactylation at K247	Promoes the DNA damage repair function of GBM, leading to chemoradiotherapy tolerance	Li et al. (2024)
	LDHA	Promoter hypermethylation leads to its aberrant expression	Promotes elevated lactate concentrations, produces acquired resistance to TAM	Hamadneh et al. (2021)
		Interacts with Beclin-1 protein	Involves in pro-survival autophagy in TAN resistant cells	Das et al. (2019)
		Catalytic generation of α-hydroxybutyric acid promotes histone H3 methylation and subsequent activation of the Wnt/β-catenin pathway	May be involves in MDR phenotyping by regulating the Wnt/β-catenin pathway	Liu et al. (2018b)
Oxidative phosphate	PKD	COL11A1 binds to PKD1, thereby inhibiting ubiquitinated degradation of PKD1 and promoting PDK1 phosphorylation of Akt	Chemotherapy resistance	Wu et al. (2015)
		HSF1 transcriptionally regulates PDK3 and prevents ubiquitination degradation of PDK3	Chemotherapy resistance	Xu et al. (2019)
	PDC	Hsp70 binds to PDC and induces PDC translocation to the nucleus. In the nucleus PDC catalyses the production of acetyl coenzyme A, which enhances the acetylation of H3K9 and H3K18 into the promoted S phase and upregulates E2F expression	E2F1 promotes ABCG2 expression, which can lead to the MDR phenotype	Sutendra et al. (2014), Rosenfeldt et al. (2014)
		PDC in the nucleus can form a protein complex with PKM2 and histone acetyltransferase p300 to acetylate H3K9	Regulation of ABCG2 gene expression	Matsuda et al. (2016)
	α-KGDH	KAT2A assists α-KGDH translocates to the nucleus and promotes histone H3 succinylation	H3K79 succinylation prevents degradation of β-conjugated proteins	Wang et al. (2017a), Tong et al. (2020)
	FH	DNA damage induces FH translocation to the nucleus and promotes NHEJ DNA repair	May be involves in MDR formation by promoting DNA damage repair	Ge et al. (2022), Jiang et al. (2015)

### 3.1 Glucose transport proteins (GLUTs)

Glucose transporters are essential for glucose uptake, ensuring glycolytic substrate availability, meeting energy requirements, preventing apoptosis, and producing increased amounts of metabolic intermediates and ATP. High levels of GLUTs in tumor biopsy samples are associated with poor prognosis in patients with cancer (Fu et al., 2020). Fourteen different GLUT isoforms have been identified, among which GLUT1, GLUT3, and GLUT4 have been the most extensively studied in the context of cancer. The upregulation of GLUT1, GLUT3, and GLUT4 is mediated by insulin and HIF-1α, and is correlated with drug resistance in cancers (Wang et al., 2021). GLUT1, the major glucose transport protein, is elevated in many cancers, including breast, cervical, colon, lung, ovarian, prostate, and thyroid cancers (Ismail and Tanasova, 2022). HIF-1α increases glucose uptake by inducing the facultative glucose transporters GLUT1 and GLUT3 in hypoxic environments (Moldogazieva et al., 2020). Hyperactivation of the Akt/mTOR/c-Myc signaling pathway in cancer cells increases the expression of GLUT4, which in turn augments the ability of multidrug resistant cells to rapidly transport and consume glucose through glycolysis (Zhang et al., 2017). Recently, it was found that the RNA demethylase human Alk B homolog 5 (ALKBH5) regulates the demethylation of GLUT4 mRNA, which causes resistance to trastuzumab and lapatinib treatment in HER2-positive breast cancer patients (Liu et al., 2022). Compared with normal neutrophils, GLUT1 expression and glycolysis are both elevated in tumor-associated neutrophils from a mouse model of lung adenocarcinoma. However, deletion of GLUT1 accelerates neutrophil turnover in tumors, attenuates tumor growth and reverses tumor chemoradiotherapy resistance (Ancey et al., 2021). GLUT1 reduced autophagy via increase of mTOR activation, which in turn stimulates GLUT1 expression, forming a positive feedback loop (Buller et al., 2008). Upregulation of GLUT1 has been found in the hypoxic regions of the human colon and breast tumors, while inhibition of GLUT1 by phloretin sensitizes cancer cells to daunorubicin-induced cytotoxicity and overcomes drug resistance under hypoxia (Cao et al., 2007). Palbociclib diminishes GLUT1 level and glucose metabolism by downregulating the Rb/E2F/c-Myc signaling in triple negative breast cancer (TNBC) (Cretella et al., 2019). These observations suggest that multidrug resistant cells are more sensitive to the alterations in GLUT1 levels and glucose transport. Paradoxically, silencing of GLUT1 has been demonstrated to attenuate cell death and potentiate chemoresistance via activation of Akt/GSK-3β/β-catenin/survivin signaling in TNBC (Oh et al., 2017). This finding implies that the potential of GlUT1 as a therapeutic target should be carefully re-evaluated.

### 3.2 Glycolytic pathway-related metabolic enzymes and MDR

#### 3.2.1 HK

**FIGURE 3:**
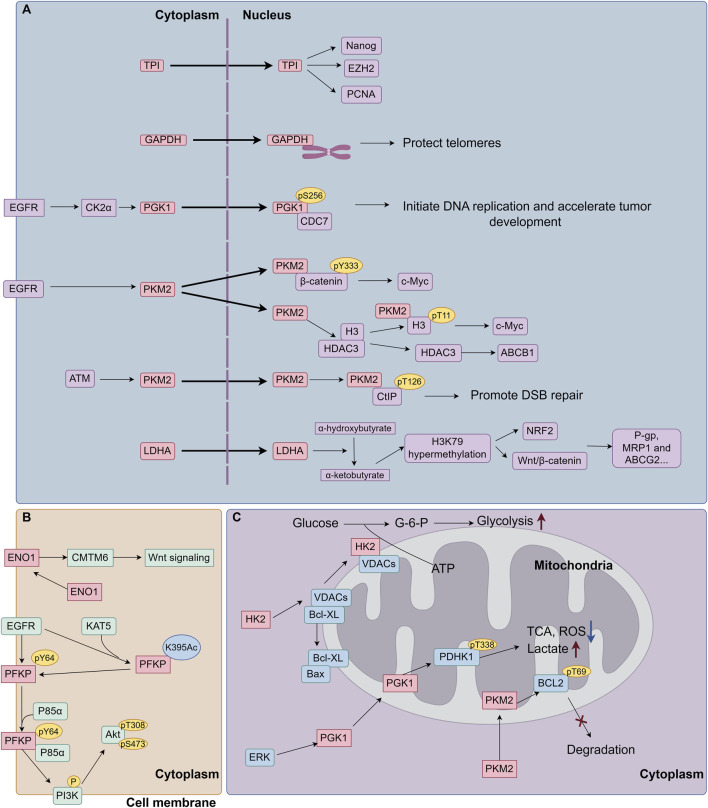
The role of aberrant localization of glycolytic enzymes on cancer MDR. **(A)** Glycolytic enzymes translocate into the nucleus. Glycolytic enzymes have the capacity to translocate from the cytoplasm to the nucleus, where they engage in non-classical function: 1) Bind to nuclear proteins (Nanog, EZH2, PCNA) to increase cancer cell resistance to chemotherapeutic drugs. 2) Protect telomeres from chemotherapy-induced degradation. 3) Function as protein kinases, to phosphorylate nuclear proteins to enhance oncogene (c-Myc) transcription expression, upregulate the expression of drug-resistance proteins (ABCB1, P-gp, MRP1and ABCG2), and promote DSB repairs. 4) Produce metabolites in the nucleus to mediate post-translational modifications of histones, such as H3K79 hypermethylation, followed by the activation of transcription factors, leading to the upregulation of MDR proteins. **(B)** Glycolytic enzymes translocate to the cell membrane. Membrane-bound ENO1 interacts with and stabilizes the CMTM6 protein, thereby activating Wnt signaling and inducing cisplatin resistance. Activated EGFR facilitates the acetylation of PFKP at K395 by KAT5, leading to its translocation to the cell membrane. Subsequently, PFKP undergoes phosphorylation at Y64 by EGFR. The phosphorylated PFKP then binds to and recruits p85α to activate PI3K/Akt signaling pathway. **(C)** Glycolytic enzymes translocate to mitochondria. HK2 can bind to mitochondrial outer membrane-localized VDACs, facilitating the rapid use of ATP newly synthesized in mitochondria for the phosphorylation of glucose. Moreover, HK competitively disrupts the association of BCL-2 family proteins with VDACs, which controls mitochondrial outer membrane permeabilization and protects tumor cells from apoptosis and chemotherapy. ERK phosphorylates PGK1 and facilitates PGK1 translocating to mitochondria, where PGK1 phosphorylates PDHK1 at T338 to reprogramming glycolysis metabolism. PKM2 has been found to translocate into mitochondria under oxidative stress, where PKM2 interacts with and phosphorylates BCL-2 at T69, preventing BCL-2 degradation and cell apoptosis. EGFR, epidermal growth factor receptor; CK2α, Casein kinase 2α; CDC7, cell division cycle 7; ATM, ataxia telangiectasia mutated; CtIP, CTBP-interacting protein; DSB, DNA double-strand break; KAT5, lysine acetyltransferase 5; VDACs, voltage-gated anion channels; PDHK1, pyruvate dehydrogenase kinase 1; CMTM6, CKLF Like MARVEL Transmembrane Domain Containing 6; ROS, Reactive Oxygen Species; TCA, Tricarboxylic Acid Cycle.

Hexokinase (HK) converts glucose to glucose-6-phosphate (G-6-P) and is the initial rate-limiting enzyme in glycolysis. Among the four HK isoforms in mammals, HK2 is a cancer-specific enzyme regulated by HIF1 and c-Myc. HK2 expression is absent or low in most normal adult cells, while it is highly expressed in cancer cells (Ciscato et al., 2021; Garcia et al., 2019). The aberrant localization of glycolytic enzymes, including their translocation to the nucleus, membrane, or mitochondria, significantly contributes to tumor progression and drug resistance. This phenomenon endows glycolytic enzymes with novel and enhanced pro-oncogenic functions (Figure 3.). HK2 can bind to mitochondrial outer membrane-localized voltage-dependent anion channels (VDACs), facilitating the rapid use of ATP newly synthesized in mitochondria for the phosphorylation of glucose. This interaction reduces the negative feedback of G-6-P on HK, which accelerates glycolysis and glucose metabolism in tumor cells (Lincet and Icard, 2015). Moreover, HK competitively disrupts the association of BCL-2 family proteins with VDACs via its N-terminal membrane binding domain, which controls mitochondrial outer membrane permeabilization and protects tumor cells from apoptosis and chemotherapy (Pastorino and Hoek, 2008). HK2 has been considered as a clinical prognostic marker in breast and liver cancers (Bao and Wong, 2021). Upregulation of HK2 can induce drug resistance in breast cancer cells (Ahmadpour et al., 2021; Sato-Tadano et al., 2013). HK can be activated through the PI3K/Akt/mTOR signaling and other survival pathways in cancer cells, resulting in MDR (Min et al., 2013). HK2 overexpression activates several oncogenic molecules, such as epidermal growth factor receptor (EGFR), Fibroblast Growth Factor Receptor (FGFFR), Akt, MEK and β-integrin (Fukushi et al., 2022). In estrogen receptor (ER)-positive breast cancer cells, HK2 suppresses mTOR activity through an interaction with mTOR, which promotes autophagy and chemoresistance to tamoxifen (Liu et al., 2019). A recent study has reported that phosphorylation of HK2 on T473 site caused by Pim-2 proto-oncogene, serine/threonine kinase (PIM2) increases HK2 protein stability and enzyme activity, and augments glucose starvation-induced autophagy. Consequently, phosphorylated HK2 assists the cell growth and paclitaxel tolerance in breast cancer (Yang et al., 2018). Unexpected activation of the Akt-mTOR pathway augments the transcription of gluconeogenesis-related metabolic enzymes, including GLUTs and HK2, which elicits a series of cascade reactions to increase ABC transporter proteins expression, proliferation and antiapoptotic capacity of leukemic cells, leading to the MDR phenotype in leukemic cells (Zhang X. et al., 2018). This study suggests that the compensatory self-protection caused by AKT-mTOR pathway is a major driver of MDR. In ovarian cancer cells, HK2 overexpression promotes the phosphorylation of ERK1/2 and MEK/ERK signaling pathway-mediated autophagy, causing chemoresistance via ATP-dependent and ABC transporter-independent mechanisms (Zhang X-Y. et al., 2018). Recent studies have found that HK2 leads to reprogramming of cellular energy metabolism by regulating the expression of ABC transporter protein and solute carrier (SLC) transporter protein genes, which in turn enhances cellular resistance to paclitaxel (Lin et al., 2024). Accumulating evidence reveals that as a self-degradative system, autophagy generally arises in response to chemotherapeutic drugs treatment and subsequently contributes to the development of MDR. Therefore, inhibition of autophagy can resensitize drug resistant cancer cells and increase the efficacy of chemotherapeutic drugs (Alexa-Stratulat et al., 2019). Considering that HK2 enables to modulate autophagy and apoptosis resistance through its canonical and noncanonical functions in tumor cells, HK2 may be a prospective candidate target for overcoming MDR. Accumulating evidence reveals that as a self-degradative system, autophagy generally arises in response to chemotherapeutic drugs treatment and subsequently contributes to the development of MDR. Therefore, inhibition of autophagy can resensitize drug resistant cancer cells and increase the efficacy of chemotherapeutic drugs (Qin et al., 2023). Considering that HK2 enables to modulate autophagy and apoptosis resistance through its canonical and noncanonical functions in tumor cells, HK2 may be a prospective candidate target for overcoming MDR.

#### 3.2.2 PGI

Phosphohexose isomerase (PGI), also called G-6-P isomerase, is a second glycolytic enzyme that catalyzes the isomerization of G-6-P to fructose 6-phosphate (F-6-P). In addition to being involved in glycolysis as a metabolic enzyme, PGI acts as an autocrine motility factor to induce the proliferation, differentiation and survival of various cancer and immune cells (Fairbank et al., 2009). The binding of PGI to human epidermal growth factor receptor 2 (HER2) activates HER2 phosphorylation and PI3K/mitogen-activated protein kinase (MAPK) signaling, leading to Akt activation. This aberrant regulation can overcome the cytotoxicity of Herceptin/Trastuzumab in breast cancer cells by activating downstream signaling pathways (Kho et al., 2013). PGI can promote drug resistance in tumor cells through the exertion of cytokine functions. The downstream target proteins of the aberrant signaling pathways that it activates are not yet known. Since the activation of Akt is associated with the expression of ABC transporter proteins, it is also worth investigating whether PGI leads to drug resistance by regulating the expression of multidrug resistance-associated transporter proteins.

#### 3.2.3 PFK1

Phosphofructokinase-1 (PFK1), the second rate-limiting enzyme in glycolysis, converts fructose 6-phosphate (F-6-P) to fructose 1,6-bisphosphate (F-1,6-BP). In human glioblastoma cells, PKF1 platelet isoform (PFKP) is the major isoform of PFK1 and functions as a key signaling molecule on the plasma membrane that regulates PI3K/Akt activation. EGFR activation induces the acetylation of PKFP by lysine acetyltransferase 5 (KAT5) at K395 and subsequently boosts the translocation of PKFP to the plasma membrane, followed by its phosphorylation at Y64 by EGFR. Phosphorylated PFKP binds to and recruits p85α to the plasma membrane, where it causes PI3K phosphorylation. As a result, PI3K-dependent AKT activation in turn enhances PFK1 activation and tumorigenesis, suggesting a positive-feedback regulation between PKFP and PI3K/Akt pathway (Lee et al., 2018). PFKP phosphorylation is required for AKT-mediated β-catenin S552 phosphorylation and subsequent β-catenin transactivation in human glioblastoma cells (Lee et al., 2020). Importantly, Akt can directly phosphorylate PFKP at S386, which prevents the interaction of PFKP with E3 ligase TRIM21 and the subsequent TRIM21-mediated polyubiquitination and degradation of PFKP, resulting in increased PFKP expression, aerobic glycolysis, cell proliferation, and tumor growth (Lee et al., 2017). Among the four isoforms of 6-phosphofructo-2-kinase/fructose-2,6-bisphosphatase (PFKFB), PFKFB3 has the highest kinase activity to converts F-6-P to fructose 2,6-bisphosphate (F2,6BP) that functions as an allosteric activator of the metabolic enzyme PFK1. The dual kinase and phosphatase activity of the PFKFB isoforms determines the content of F2,6BP. The lactate generated in cells inhibits PFK1 activity by changing the structural conformation of PFK1, while F2,6BP stabilizes the PFK1 enzyme via its metastable activation and conformational shift in the presence of lactate. PFKFB3 is frequently upregulated in a variety of cancers, such as breast, colon, pancreatic, and prostate cancers (Jones et al., 2022). Thus, PFKFB3 inhibition has been considered as a compelling therapeutic strategy in glucose-dependent cancers. A recent study reported that PFKFB3 was mainly located in the nucleus and assisted the DNA repair and apoptosis resistance through Akt/ERCC1 signaling, resulting in the failure of chemotherapy and radiotherapy in HCC cells (Shi et al., 2018). Emerging evidence has suggested that PFKFB3 is essential for maintaining glycolysis and promoting metabolic reprogramming in tumorigenic environments, which triggers cell proliferation, anchorage-independent growth, vascular invasiveness, reactive oxygen species (ROS) detoxification, metastasis and drug resistance even under unfavorable conditions (Galindo et al., 2022; Xintaropoulou et al., 2018; Shi et al., 2017). PFKFB3 phosphorylation is increased in highly drug resistance cell lines, accompanied by the elevated level of glycolytic activity. Blocking the active form of PFKFB3^S461^ by a novel inhibitor PFK158 leads to decreased glucose uptake, lactate production, and ATP levels in gynecologic cancer cells and restores the chemosensitivity of cancer cells to carboplatin and paclitaxel (Mondal et al., 2019). In addition, the upregulation of PFKFB3 was observed in MDR breast cancer cells (Sengupta et al., 2018). PFKFB4 has a noncanonical function in the nucleus, where it binds to and phosphorylates steroid receptor cofactor-3 (SRC-3) at S857. Phosphorylated SRC-3 recruits transcription factor ATF4 to the promoters of adenosine monophosphate deaminase-1 (AMPD1) and xanthine dehydrogenase (XDH) genes, leading to increased expression of AMPD1 and XDH that drives purine synthesis and enables glucose flux towards the pentose phosphate pathway (Dasgupta et al., 2018). Because purine synthesis facilitates the repair of DNA damage caused by chemotherapeutic agents in cancer cells, PFKFB4 has been considered to promote the tolerance to cytotoxic drug. Tumor cells resist the cytotoxicity of therapeutic drugs by inhibiting apoptosis and promoting DNA damage repair, leading to the development of multidrug resistance. In addition to their metabolic enzyme functions, the PFK and PFKFB proteins are involved in the regulation of apoptosis and DNA repair. Aberrant expression of these proteins has been detected in a variety of MDR cells. Therefore, further investigation of whether nonglycolytic functions of the PFK and PFKFB proteins are directly involved in the development of multidrug resistance is important for the discovery of new mechanisms of MDR development as well as the development of new targeted therapeutic agents.

#### 3.2.4 TPI

Triosephosphate isomerase (TPI) is a glycolytic enzyme that catalyzes the interconversion of dihydroxyacetone phosphate (DHAP) and glyceraldehyde 3-phosphate (G-3-P). Recent evidence has reported that the TPI level is increased and is associated with patient prognosis in lung cancer, and urothelial carcinoma (Lincet and Icard, 2015). TPI has become recognized as a tumor biomarker in human gastric and lung squamous cell carcinomas and is linked to tumor progression as well as drug resistance (Lone et al., 2018). High expression of TPI1 is an unfavorable factor of overall survival (OS) in patients with lung adenocarcinoma (LUAD). In contrast, TPI has been reported to function as a tumor suppressor driven by the β-catenin/p53 signaling axis in HCC (Fukushi et al., 2022). A recent study revealed that the oncogenic function of TPI in LUAD was dependent on its translocation to the nucleus rather than on its catalytic activity, where it interacts with nuclear proteins such as Nanog, EZH2, or PCNA to form nuclear complexes (Liu et al., 2022). TPI have been found to translocate to the nucleus to promote drug resistance in tumor cells, in addition to functioning as glycolytic enzymes in the cytoplasm (Yang et al., 2024). TPI interacts with the long noncoding RNA Linc00942, triggering phosphorylation and dimerization of TPI for translocation into the nucleus, thereby inhibiting HDAC3 activity, promoting high-mobility group box (HMG-box) factor SOC9 expression and promoting temozolomide resistance in glioblastoma cells (Yang et al., 2024). In breast cancer cells, TPI is highly upregulated and activates the PI3K/Akt/mTOR pathway to promote tumor progression. The hypoxic environment can induce glucose metabolism through HIF, which can lead to drug resistance in tumor cells (Bao and Wong, 2021). Hypoxia is an important feature of solid tumors, and in hepatocellular carcinoma and pancreatic ductal adenocarcinoma hypoxia induces TPI upregulation (Bao and Wong, 2021; Sun et al., 2021). Oxidative stress and exposure to chemotherapeutic drugs stimulate the nuclear translocation of TPI and increase cancer cell resistance to chemotherapeutic drugs. STK11/LKB1 inactivating mutations often cooccur with KRAS-activating mutations, and STK11/LKB1 inactivating mutations are also a driver of primary resistance to immunotherapy in KRAS-mutant lung adenocarcinoma (LUAD) (Qian et al., 2023). However, the underlying mechanism still needs to be further studied and elucidated. TPI activity in LUAD can be regulated by salt-induced kinase (SIK) in an LKB1-dependent manner (Stein et al., 2023). Given that the high expression of TPI is involved in a variety of pathways associated with the MDR phenotype, it is worth exploring whether TPI induces MDR phenotypes in tumors through nuclear translocation and is involved in the HIF-related drug resistance pathway in an LKB1-dependent manner.

#### 3.2.5 GAPDH

Glyceraldehyde-3-phosphate dehydrogenase (GAPDH) converts G-3-P to 1,3-bisphosphoglycerate (1,3-BPG), the first step in the production of NADH during glycolysis, and has been confirmed to participate in DNA repair, tRNA transport, iron metabolism, membrane transport, histone biosynthesis, the maintenance of DNA integrity, and receptor-mediated cell signaling, beyond its glycolytic function. Overexpression of GAPDH is a characteristic associated with accelerated proliferation in many tumors, such as breast, lung, pancreatic, and esophageal tumors, suggesting GAPDH as an attractive target for the treatment of tumors (Li et al., 2020). Apart from its metabolic role, GAPDH also exhibits noncanonical functions to promote MDR. For example, the increased nuclear localization of GAPDH was observed in A549 cells exposed to gemcitabine and doxorubicin, and GAPDH bound to telomeric DNA to protect telomeres from chemotherapy-induced degradation and growth inhibition (Demarse et al., 2009). A recent study reveals a regulatory effect of GAPDH on mTOR activity. Under low-glucose conditions, GAPDH binds to Rheb, a small GTPase that is a key proximal activator of mTORC1, and subsequently prevents the association of Rheb and mTOR, leading to the inhibition of mTOR. High glycolytic flux blocks the GAPDH/Rheb interaction, thus enabling signal transduction through the mTOR pathway (Lee et al., 2009). Considering the critical effect of mTOR signaling on MDR, we infer that GAPDH may be involved in the induction of MDR by coordinating mTOR activity. Markus and colleagues discovered that yeast cells counteracted the deleterious oxidative stress through inactivation of GAPDH, which caused a redirection of the metabolic flux from glycolysis to the PPP, followed by the increased cytoplasmic NADP(H) pool and maintenance of cellular redox equilibrium (Ralser et al., 2007). In addition, GAPDH along with aldolase and phosphofructokinase are organized into complexes on human erythrocyte membrane, where allows the direct consumption of ATP by ion pumps without release into the cytoplasm (Campanella et al., 2005). This role of GAPDH is consistent with its paracrine function in drug efflux. Therefore, whether the nonmetabolic function of GAPDH contributes to the development of MDR needs to be further investigated.

#### 3.2.6 PGK1

Phosphoglycerate kinase 1 (PGK1) catalyzes the conversion of 1,3-bisphosphoglycerate (1,3-BPG) to 3-phosphoglycerate (3-PGA) and thus producing the first ATP molecule in the glycolytic pathway. PGK1 has been found to be overexpressed in a variety of tumors, including breast cancer and pancreatic ductal adenocarcinoma (PDAC), as well as in multidrug resistant ovarian cancer cells (Qiu et al., 2024; Chen et al., 2015). PGK1 confers sorafenib resistance by enhancing CXCR4-mediated ERK phosphorylation and glycolysis in renal clear cell carcinoma (He et al., 2022). NSCLC cells with high PGK1 expression exhibit epithelial-mesenchymal transition remodeling and less response to erlotinib treatment (Cheng et al., 2020). Increasing evidence indicates that PGK1 functions as a protein kinase other than metabolic enzymes of glycolysis. For instance, oncogenes activation induces ERK-dependent PGK1 phosphorylation at S203 and subsequently facilitates the translocation of PGK1 to mitochondria, where PGK1 phosphorylates pyruvate dehydrogenase kinase 1 (PDHK1) at T338. This inhibits pyruvate utilization in tricarboxylic acid (TCA) cycle and ROS production, while promotes lactate production and tumorigenesis in glioblastoma, highlighting a noteworthy role of PGK1 in coordinating glycolysis and the TCA cycle (Li et al., 2016). The mitochondrial accumulation and activity of PGK1 can be stimulated through reversible and dynamic O-linked N-acetylglucosamine (O-GlcNAc) modification at T255, which is independent of S203 phosphorylation (Nie et al., 2020). Casein kinase 2α phosphorylates nuclear PGK 1 at S256 in response to oncogenic EGFR signaling, leading to interaction between PGK1 and cell division cycle 7 (CDC7). CDC7-bound PGK1 rescues the assembly and activity of CDC7 complex and recruits DNA helicase to initiate DNA replication and accelerate tumor development (Li et al., 2018). Moreover, PGK1 can be acetylated by acetyl-transferase ARD1 at K388 in glutamine deprivation and hypoxia condition, followed by PGK1-mediated Beclin1 S30 phosphorylation that is indispensable for autophagy induction and homeostasis maintenance (Qian et al., 2017). A recent study verifies that mitochondrial PGK1 drives the self-renewal of liver tumor-initiating cells (Chen et al., 2023). The above nonmetabolic functions of PGK1 suggest that PGK1 contributes to MDR by eliciting lactate production, DNA damage repair, autophagy, and stem cell characteristics.

#### 3.2.7 PGAM

Phosphoglycerate mutase (PGAM) is an important glycolytic enzyme that catalyzes the interconversion of 3-PGA and 2-phosphoglycerate (2-PGA). PGAM expression is abnormally high in several human cancers and inhibition of PGAM is lethal to cancer cells (Evans et al., 2005). In addition to its metabolic functions, PGAM can also increase the stability of CTBP-interacting protein (CtIP) in the nucleus and assist homologous recombination repair of DNA double-strand breaks caused by chemotherapy agents camptothecin and cisplatin in cancer cells. In contrast, enzymatic inhibition of PGAM1 impairs the intracellular deoxyribonucleotide triphosphate pool and sensitizes BRCA1/2-proficient breast cancer to poly(ADP-ribose) polymerase (PARP) inhibitors (Qu et al., 2017). Shen and colleagues identified a novel PGAM1 allosteric inhibitor HKB99 that blocked conformational change of PGAM1 during catalytic process, and demonstrated an excellent impact of HKB99 for improving erlotinib resistance through alterations of multiple signaling pathways in NSCLC (Huang K. et al., 2021). High level of PGAM1 was found in the paclitaxel resistant ovarian cancer cells, accompanied by increased pyruvic acid or lactic acid production (Feng et al., 2022). Moreover, targeting PGAM2 reversed the tolerance to androgen receptor inhibitor enzalutamide in castration resistant prostate cancer by suppressing BCL-2 pathway (Li et al., 2023).

#### 3.2.8 ENO

Enolase (ENO) catalyzes the dehydration of 2-phosphoenolpyruvate (2-PGA) to phosphoenolpyruvate (PEP). There are three isoforms of ENO in mammals: ENO1 (α-enolase), ENO2 (γ-enolase) and ENO3 (β-enolase). ENO1 is ubiquitously expressed in most adult mammal tissues, while ENO2 and ENO3 are primarily restricted to neural and skeletal muscle cells, respectively. Therefore, ENO2 is also termed as neuron-specific enolase (NSE) and ENO3 is called muscle-specific enolase (MSE). A pervious study revealed that ENO1 was reversely correlated with the prognosis of patients with Non-Hodgkin’s Lymphomas and promoted cell adhesion mediated drug resistance (Zhu et al., 2015). Notably, upregulated expression of ENO1 was verified to participate in multiple drug resistance in methotrexate resistant human breast cancer cells (Chen et al., 2014). The methylation of ENO1 caused by arginine methyltransferase 6 facilitates the generation of active ENO1 dimers and its binding affinity to 2-PGA, resulting in increased tumor growth and cisplatin tolerance in lung cancer (Sun et al., 2023). Consistently, Mohapatra and colleagues demonstrated that membrane-bound ENO1 interacted with CKLF Like MARVEL Transmembrane Domain Containing 6 (CMTM6) and subsequently stabilized CMTM6 protein, which contributed to cisplatin resistance via activation of Wnt signaling in oral squamous cell carcinoma (Mohapatra et al., 2021). The expression and surface localization of ENO1 were positively associated with the cancer progression, invasiveness and doxorubicin-resistance in breast cancer (Perconti et al., 2017). In contrast, downregulation of ENO1 by specific small interfering RNA effectively restored 4-hydroxytamoxifen-induced cytotoxicity in tamoxifen resistant breast cancer cells (Tu et al., 2010). Depletion of ENO1 attenuated 5-FU resistance through inhibition of epithelial-mesenchymal transformation process in colorectal cancer (Gu et al., 2022). Besides ENO1, ENO2 also has been involved in drug resistance. For instance, ENO2 augmented cell proliferation and glucocorticoid resistance by increasing expression of various glycolysis-related genes in acute lymphoblastic leukemia (Liu CC. et al., 2018). ENO2 alters glycolytic flux from mitochondrial OXPHOS to glycolysis, which continuously maintaining the competitive advantage of lenvatinib-resistant HCC cells over sensitive HCC cells (Wang et al., 2024). Importantly, ENO2-derived metabolite phosphoenolpyruvate functioned as a selective inhibitor of HDAC1, which activated the β-catenin signaling and desensitized colorectal cancer to antiangiogenic drugs (Wang C. et al., 2023). Based on the above evidence, blocking the expression and activity of ENO1/2 may provide a potential therapeutic strategy to reverse drug resistance.

#### 3.2.9 PKM2

Pyruvate kinase (PK) is the rate-limiting enzyme in the final step of glycolysis and catalyzes an irreversible transphosphorylation reaction between PEP and ADP to form pyruvate and ATP. PK has four different isoforms: L, R, M1, and M2. The L (PKL) and R (PKR) isoforms are expressed in liver cells and red blood cells, respectively. PKM gene transcription results in the generation of two distinct isoforms (PKM1 and PKM2) by alternative splicing to control the mutually exclusive inclusion of exon 9 or 10, respectively. As a key regulatory protein of tumor metabolism, PKM2 is highly expressed in many tumors and promotes tumor cell proliferation and metastasis. In normally differentiated cells, PKM2 alternates between a highly active tetrameric form and a less active dimeric form. In tumor cells, PKM2 is present mainly as the low-activity dimeric form, which leads to increased accumulation of diverse glycolytic intermediates required for macromolecule biosynthesis and thus promotes cancer proliferation (Zhao et al., 2022). The functions of PKM2 differ between the cytoplasm and nucleus. Cytoplasmic PKM2 interacts with CD44 (a cell surface biomarker of cancer stem cells) and boosts glucose uptake and PPP flux, which maintains cellular redox homeostasis and protects cancer cells against anticancer drugs (Tamada et al., 2012). The NADPH oxidase isoform NOX4, located in the inner mitochondrial membrane, induced the production of ROS, which inhibited p300/CBP-associated factor-dependent acetylation and lysosomal degradation of PKM2. This induces the generation of etoposide resistance in both *in vivo* and *in vitro* in the renal cell carcinoma model (Shanmugasundaram et al., 2017). EGFR activation promotes translocation of PKM2 into the nucleus, where PKM2 binds to and transactivates β-catenin, leading to elevated transcription of β-catenin target gene c-Myc that contributes to MDR (Yang et al., 2017; Yang et al., 2012). Importantly, nuclear PKM2 also exhibits protein kinase activity to phosphorylate histone H3 at T11, which is essential for the dissociation of HDAC3 from the c-Myc promoter to initiate c-Myc transcription (Yang et al., 2014). Because reduced HDAC3 activity is associated with ABCB1 upregulation in lung cancer (Wang H. et al., 2023). This finding implies that PKM2 may be involved in MDR phenotyping by regulating ABCB1 expression through an epigenetic modification mechanism. PKM2 acts as an intranuclear protein kinase that phosphorylates the STAT3 protein in addition to H3, thus promoting the expression of its downstream genes (Wang B. et al., 2018). In glioblastoma, PKM2 phosphorylates STAT3, thereby promoting SOX9 expression and TMZ resistance with the assistance of TPI within the nucleus (Yang et al., 2024). Upon exposure to ionizing radiation, ataxia telangiectasia mutated (ATM)-dependent PKM2 T328 phosphorylation is sufficient to assist nuclear accumulation of PKM2 and subsequent phosphorylation of CtIP at T126, resulting in DNA double-strand break (DSB) repair via homologous recombination (HR) and tolerance to various DNA-damaging drugs (Sizemore et al., 2018). High level of nuclear PKM2 is involved in TAM and gemcitabine resistance in breast cancer and pancreatic cancer, respectively (Ge et al., 2019; Kim et al., 2015). Notably, chronic exposure of chemotherapy agents enables to induce PKM2 expression via different mechanisms (Calabretta et al., 2016; Martin et al., 2020; Yu et al., 2020), suggesting a positive feedback between PKM2 and drug resistance. Under oxidative stress, PKM2 has been found to translocate into mitochondria, where PKM2 interacts with BCL-2 and phosphorylates it at T69, preventing Cul3-based E3 ligase for BCL-2 degradation and consequent cell apoptosis in glioblastoma (Liang et al., 2017). Several studies have shown that PKM2 translocates to the nucleus when it is stimulated by different signaling pathways in a variety of tumor cells. Instead of catalyzing the production of pyruvate as a metabolic enzyme in glycolysis, it functions as a protein kinase, directly regulating gene expression, DNA repair, and apoptosis. The pathways regulated by PKM2 in the nucleus are often associated with the mechanism of MDR phenotype formation, and some studies have also shown that the nonmetabolic enzyme function of PKM2 can promote drug resistance in tumor cells. Therefore, the role of the versatility of PKM2 in the development of MDR and the related mechanisms are worth exploring. PKM2 may be a feasible and promising target for overcoming MDR in cancer cells.

#### 3.2.10 LDH

Lactate dehydrogenase (LDH) catalyzes the conversion of pyruvate to lactate and is highly studied for its role at the intersection of the glycolytic pathway and the OXPHOS pathway. LDH has two main isozymes, LDHA and LDHB, which are overexpressed in tumor cells. Aberrant LDHA and LDHB expression due to hypomethylation of the promoter and the resulting increased lactate concentration are involved in acquired TAM resistance in breast cancer cells (Hamadneh et al., 2021). LDHA was associated with Beclin-1 to provoke pro-survival autophagy in TAM resistant breast cancer (Das et al., 2019). Elevated level and activity of LDHA is observed in K562/MDR leukaemia cells, and oxamate (an LDHA inhibitor) not only inhibits glycolytic flux but also restores the sensitivity of K562/MDR cells to adriamycin by counteracting Akt/mTOR/c-Myc signaling (Zhang et al., 2017). Yang et al. reported that LDHA was one of three biomarkers for discriminating between trastuzumab resistant and responsive patients with breast cancer (Yang et al., 2020). Specific inhibition or knockdown of LDHA is able to delay tumor growth and rescue sensitivity of chemotherapy resistance in multiple myeloma (Maiso et al., 2015). A recent study revealed that the acquired resistance to FK866 was due to a shift towards a glycolytic metabolism-mediated by LDHA activity but not dependent on NAMPT mutations (Thongon et al., 2018). Human papillomavirus infection triggered oxidative stress promotes the entry of LDHA into the nucleus, where LDHA exhibited a non-canonical enzyme activity to produce α-hydroxybutyrate from α-ketobutyrate and promoted histone H3K79 hypermethylation, followed by the activation of Nrf2-mediated antioxidant responses and Wnt/β-catenin pathway (Liu Y. et al., 2018). Nrf2 and Wnt/β-catenin signaling have been demonstrated to increase transcript and protein levels of multiple ABC transporter protein (including P-gp, MRP1 and ABCG2) and confer chemoresistance phenotype in various cancers (Kukal et al., 2021; Hemmati-Dinarvand et al., 2022; Cui et al., 2018; Kim et al., 2020). These findings suggest that LDHA may contribute to MDR via modulation of Wnt/β-catenin pathway.

### 3.3 Glycolytic metabolites and MDR

Emerging evidence unveils that the products of glycolysis, such as ATP, pyruvate, and lactate, also play important roles in the development of the MDR phenotype. For example, intracellular ATP levels are a fundamental determinant of MDR in colon cancer cells, whereas depletion of ATP cause cross resistant cells to become resensitized to multiple chemotherapeutic drugs (Zhou et al., 2012). Although the rate of ATP production via glycolysis is lower than that via OXPHOS, which may limit the availability of ATP for use by ABC transporter proteins, the continuous flux of glucose ensures constant ATP synthesis. An uninterrupted supply of ATP ensures that ABC transporter proteins continuously export chemotherapeutic drugs from the cell to protect against drug toxicity. Recent studies have shown that drug efflux is significantly reduced when ATP depletion is induced. Moreover, the intracellular drug concentrations increased. This helps to overcome multidrug resistance (Wang J. et al., 2023). Therefore, targeting metabolic enzymes in the glycolytic pathway to regulate the intracellular ATP content may be an effective strategy to overcome MDR.

The glycolytic rate is generally increased in multidrug resistant cancer cells, accompanied by robust production of lactate, which is exported extracellularly and facilitates the maintenance of an alkaline intracellular pH as well as the acidic TME required for the survival of multidrug resistant cells (Icard et al., 2018). The alkaline environment of the cytoplasm leads to histone acetylation, gene expression and increased protein synthesis, whereas the acidic conditions in the TME contribute to maximal P-gp catalytic activity (Assaraf et al., 2019). Moreover, the acidic TME enables to build a chemical barrier that prevents the entrance of certain chemotherapeutic agents into cells via passive diffusion. Treatment with proton pump inhibitors (PPIs) can result in a lower intercellular pH and a higher extracellular pH, which can increase the efficacy of chemotherapeutic drugs in multidrug resistant tumors (Taylor et al., 2015). Recently, lactate has been shown to drive an important post-translational modification called lactylation on histone lysine residues that stimulates gene transcription (Zhang et al., 2019). The lactylation is associated with epigenomic and metabolic reprogramming, inflammation, DNA damage repair, and cell differentiation etc (Wang T. et al., 2023). Recent studies have found that ALDH1A3 interacted with PKM2 and facilitated PKM2 tetramerization and lactate accumulation, following by inducing the lactylation of X-ray cross complementing protein 1 (XRCC1) at K247 in glioblastoma (GBM). Moreover, lactylated XRCC1 enhanced its translocation to the nucleus, thereby augmenting the DNA repair response to chemoradiotherapy (Li et al., 2024). Whether the increase in the lactic acid content caused by glycolysis in multidrug resistant cells can directly induce lactylation on ABC transporter or MDR-related proteins requires further investigation. Lactate is the most prominent product of the glycolytic pathway and the important role of lactate in cancer progression has been confirmed by many studies. In recent years, the first discovery of lactation modification has also highlighted the important role of lactate in the development of tumorigenesis. Research on lactation modification has revealed that lactate can promote tumor progression through epigenetic regulation, especially that of histones. Recent studies have demonstrated that MDR cells promote lactate production by increasing glycolytic activity. The accumulated lactate regulates the lactylation of H3 at the K14 site to upregulate downstream target gene expression, thereby promoting the malignant progression of MDR cells (Zeng et al., 2025). At present, there are few studies on the effect of lactation modification on the formation of MDR. Given glycolysis and the important role of lactate in MDR cells, It is of great significance to explore the relationship between glycolysis and lactation modification and the malignant progression of MDR. Reversing aberrant lactation modifications by targeting glycolytic pathways to restore drug sensitivity in cells may be a highly effective strategy.

Pyruvate levels are associated with increased P-gp expression and efflux of the P-gp substrate in solid tumors. Pyruvate serves as an antioxidant to scavenge intracellular ROS from the mitochondrial respiratory chain and contributes to the multidrug resistance phenotype (Wartenberg et al., 2010). Low levels of ROS can promote the self-renewal of cancer stem cells and play a role in resistance therapy (Huang H. et al., 2021). Increasing cellular ROS levels to treat MDR has been shown to be a viable strategy (Wang J. et al., 2023). Furthermore, Mungo and colleagues reported that treatment with pyruvate improves respiratory chain function, reduces MRP1 activity, and increases the efficacy of chemotherapy in drug-resistant cancer cells (Mungo et al., 2018).

Multiple products of the glycolytic pathway are important for maintaining multidrug resistance in tumors. Metabolic enzymes in the glycolytic pathway act as “controllers” that regulate the production of these metabolites. Therefore, targeting specific metabolic enzymes to regulate metabolite content is also a promising strategy for the treatment of MDR. Regulating the levels of these metabolites in cells by other means has also been shown to be an effective strategy for the treatment of MDR. Different cancers have different preferences for treatment.

## 4 OXPHOS and MDR

Emerging evidence indicates that energy-dependent ABC transport proteins are overexpressed due to the elevated ATP level in multidrug resistant cells, which increases the functionality of ABC transport proteins, thereby impeding the accumulation of anticancer drugs and fostering drug resistance. OXPHOS produces more ATP but is slower than glycolysis. Glycolysis facilitates the rapid removal of strong drugs from cancer cells, while OXPHOS is indispensable for providing a sufficient supply of ATP to ABC transport proteins in cancer cells with prolonged exposure to chemotherapeutic drugs. Higher concentrations of doxorubicin were found to result in elevated OXPHOS activity, upregulation of P-gp, and drug resistance in osteosarcoma cells (Buondonno et al., 2016). Although aerobic glycolysis is significantly promoted in tumor cells, particularly in multidrug resistant cells, most malignant cells still rely heavily on OXPHOS as their primary energy source (Altieri, 2019). Metabolic shift toward OXPHOS are considered a distinctive metabolic trait of drug resistance in cancer, while targeting OXPHOS abates resistance to 5-fluorouracil in colon cancer, docetaxel in prostate cancer, MAPK inhibitor in melanoma, and EGFR-TKI in EGFR-driven lung adenocarcinoma (Bosc et al., 2017). In addition, suppressing OXPHOS and depleting ATP can hinder the function of MDR transporters to continuously export drugs and restores sensitivity in chemoresistant cells (Zhao et al., 2023). Cancer cells utilize various oncogenic signaling pathways to activate OXPHOS to gain a survival advantage during anticancer therapy, thus posing a considerable therapeutic challenge.

### 4.1 TCA cycle metabolic enzymes and MDR

#### 4.1.1 PDH

Pyruvate dehydrogenase (PDH), a component of the pyruvate dehydrogenase complex (PDC), is a rate-limiting enzyme in the first step of OXPHOS in cells. It acts as a “gatekeeper” to convert pyruvate to acetyl coenzyme A (acetyl-CoA), which participates in the TCA cycle. Pyruvate dehydrogenase kinase (PDK) phosphorylates and deactivates PDH, followed by the entrance of pyruvate to the lactic acid cycle. Therefore, inhibition of PDK is an attractive strategy for anticancer therapy. PDK family proteins predominantly locate within the mitochondrial matrix and have four members with distinct tissue or cell type specificity. PDK1 is highly expressed in skeletal muscles, islets, and heart. PDK1 is a precursor enzyme that determines the fate of pyruvate and plays a key role in the regulation of mitochondrial activity. Elevated levels of cell surface PDK1 have been observed in aggressive cancer types, including lung cancer, gastric cancer, and myeloma (Anwar et al., 2021). Collagen type XI alpha 1 (COL11A1) has been identified a chemotherapy response-associated gene in epithelial ovarian carcinoma. Cisplatin or paclitaxel administration enhances the interaction between PDK1 and COL11A1 and subsequently attenuates the ubiquitination and degradation of PDK1, leading to activation of PDK1-mediated Akt phosphorylation and chemoresistance (Wu et al., 2015). PDK2 is ubiquitously distributed in diverse tissues, except for the lung and spleen. PDK2 is upregulated in paclitaxel- or cisplatin resistant lung cancer cells compared to the parental cells and is associated with poor prognosis in patients with lung cancer (Sun et al., 2017; Hu et al., 2019). Combination treatment with paclitaxel and a PDK inhibitor (dichloroacetate (DCA)) was found to substantially suppress the proliferation of paclitaxel resistant lung cancer cells. PDK3 is limited to the brain, kidneys, and testes. Chemical or genetic inhibition of PDK3 was found to improve the sensitivity of resistant cells to treatment. Heat shock factor 1 (HSF1) binds to and stabilizes PDK3 protein, forming a positive feedback loop that promotes glycolysis and chemoresistance in cancer cells (Xu et al., 2019). PDK4 is expressed primarily in liver, muscle, and certain epithelial cells. Upregulation of PDK4 is observed in high-grade invasive bladder cancers compared with low-grade bladder cancers, and Knockdown or inactivation of PDK4 delays cell proliferation and sensitizes bladder cancer cells to cisplatin (Woolbright et al., 2018). In osteosarcoma, miR15b-5p targeted PDK4 and decreased its expression to exert tumor-suppressive effects by blocking aerobic glycolysis (Weng et al., 2018).

#### 4.1.2 PDC

Sutendra *et al.* reported that in addition to its canonical function as a metabolic enzyme in the mitochondrial TCA, the PDC was able to translocate to the nucleus to during cell-cycle progression, producing a nuclear pool of acetyl-CoA from pyruvate and promoting histone acetylation and E2F1 expression (Sutendra et al., 2014). E2F1, as a bHLH transcription factor, can directly bind to the ABCG2 gene promoter and increase its expression (Rosenfeldt et al., 2014), suggesting a potential role of PDC on the development of the MDR phenotype. A recent study discovered a nuclear acetyl-CoA production system in which PKM2 and PDC constituted a complex to locally provide acetyl-CoA to acetyltransferase p300 for histone H3K9 acetylation at the gene enhancer, leading to the enhancement of AhR-mediated detoxification to chemotherapeutic drugs in tumor cells (Matsuda et al., 2016). Moreover, histone H3K9 acetylation has been demonstrate to induce ABCG2 gene transcription in multidrug resistant cells (To et al., 2008), further supporting that the nuclear translocation of PDC may confer MDR in cancer cells.

#### 4.1.3 α-KGDH

α-Ketoglutarate dehydrogenase (α-KGDH) catalyzes the generation of succinyl CoA, CO_2_ and NADH from α-ketoglutarate, NAD^+^ and CoA. In addition to functioning as a metabolic enzyme in the TCA cycle, α-KGDH has been shown to localize in the nucleus, where it binds to acetyltransferase KAT2A in the promoter regions of target genes and subsequently facilitates KAT2A-mediated histone H3K79 succinylation, resulting in increased gene transcription, cell proliferation and tumor growth (Wang Y. et al., 2017). KAT2A-induced H3K79 succinylation upregulates 14-3-3ζ expression, followed by enhanced β-catenin stability and the expression of β-catenin target genes (including cyclin D1, c-Myc, GLUT1, and LDHA), which in turn promotes glycolysis and malignant progression of pancreatic carcinoma (Tong et al., 2020). Although there is no direct evidence that α-KGDH is involved in drug resistance, we infer that α-KGDH may contribute to MDR via KAT2A-mediated histone succinylation in tumor cells.

#### 4.1.4 FH

Fumarate hydratase (FH) is a key enzyme involved in the TCA cycle that catalyzes the reversible interconversion of fumarate to malate via hydration and dehydration. FH acts as a double-edged sword in drug resistance. On the one hand, FH deficiency causes the accumulation of oncometabolite fumarate that inactivates PTEN by directly reacting with PTEN at C211 to form S-(2-succino)-cysteine and ultimately sensitizes human type2 papillary renal cell carcinoma to sunitinib (Ge et al., 2022). On the other hand, cytosolic FH as a component of the DNA damage response shuttles into the nucleus upon ionizing radiation and protects cells against DNA damage, suggesting that FH may participate in MDR development via promotion of DNA damage repair (Jiang et al., 2015). However, the detailed function of FH in MDR needs further investigation.

### 4.2 OXPHOS-related metabolic pathways and MDR

The role of mitochondrial function in tumors has historically been overlooked due to the discovery of the Warburg effect. Mitochondrial gene mutations have been shown to confer a growth advantage during tumor development. Although most cancer cells harbor mutations in mitochondrial genes, these mutations cannot completely abolish the metabolic function of mitochondria. Mitochondrial dysfunction triggers the activation of signaling pathways connecting mitochondria and the nucleus in cancer cells, inducing the expression of specific transcription factors. This ultimately alters the expression of proteins associated with apoptosis resistance, cancer cell growth and energy production. Different cancers exhibit distinct profiles of metabolic reprogramming, which is partly dependent on glycolysis and partly dependent on OXPHOS. Due to intratumor heterogeneity, cancer cells with different metabolic profiles may coexist and coordinate in the same tumor, which is referred to as metabolic symbiosis. For example, simultaneous stimulation of glycolysis and OXPHOS was found in melanoma cells (Bosc et al., 2017), with important implications for melanoma development. Treatment of melanoma cells with the glycolysis inhibitor 2-deoxy-D-glucose (2-DG) induces a metabolic shift to OXPHOS for energy production. Metabolic symbiosis provides a sound basis for the development of acquired drug resistance by endowing tumor cells with metabolic plasticity. Peroxisome proliferator-activated receptor gamma (PPARγ) coactivator 1 alpha (PGC1α), a transcriptional coactivator of mitochondria-related genes, is involved in the regulation of mitochondrial biosynthesis and governs many pathways associated with therapeutic resistance, including OXPHOS, the oxidative stress response, glutamine metabolism and glutathione metabolism (Lv et al., 2022). Gentric *et al.* revealed metabolic heterogeneity in high-grade serous ovarian cancer (HGSOC), which could be divided into low and high OXPHOS subgroups. Low OXPHOS HGSOCs prefer to a glycolytic metabolism, but high OXPHOS tumors exhibit optimized mitochondrial respiration supported by fatty acid and glutamine oxidation, and are subject to chronic oxidative stress. Notably, increased response to conventional chemotherapies is observed in high-OXPHOS HGSOCs, highlighting a link between OXPHOS status and drug resistance (Gentric et al., 2019). SMARCA4 is the most frequently inactivated subunits of the SWI/SNF complex in lung adenocarcinoma. SMARCA4 mutant cells have elevated mitochondrial DNA content, oxygen consumption and respiratory capacity, and are sensitive to OXPHOS inhibitor IACS-010759 by blunting transcriptional response to energy stress (Deribe et al., 2018). The resistance of BRAF and NRAS mutated melanoma cells to the MEK inhibitor selumetinib is also associated with the increased level of OXHPOS activity (Gopal et al., 2014). In colon cancer cells, numerous genes associated with OXPHOS and mitochondrial biogenesis are significantly upregulated upon exposure to chemotherapy, which is mediated by deacetylase sirtuin-1 and its substrate, the transcriptional coactivator PGC1α (Vellinga et al., 2015). Moreover, active OXPHOS results in upregulation of P-gp/ABCB1, MRP1/ABCC1, MRP5/ABCC5, and BCRP/ABCG2 in cancer cells with p53 mutations or p53 allelic deletion, while the opposite phenomenon is observed in cells with wild type P53 (Belkahla et al., 2018). OXPHOS-derived ROS significantly enhances the mRNA levels of ABCC2 and ABCG2 through the Nrf2/Keap1 system, while the induction of ABCC1 transcription by ROS may involve an Nrf2-independent mechanism (Alexa-Stratulat et al., 2019). Consistently, oxidative stress promotes the expression and activity of HIF-1α, which in turn binds directly to the promoters of genes encoding P-gp to stimulate P-gp transcription and facilitate the acquisition of the MDR phenotype in tumor cells (Seebacher et al., 2015). Increased level of OXPHOS is found in chemoresistant cells compared with their sensitive counterpart, accompanied by elevated production of proinflammatory cytokine interleukin 6 (IL-6). IL-6 selectively induces P-gp and confers ovarian cancer cells resistance to cisplatin-triggered apoptosis (Matassa et al., 2016). These findings indicate that blocking OXHPOS may be a feasible strategy for alleviating tumor resistance to increase the antitumor effect of chemotherapy.

## 5 The PPP and MDR

During PPP, G-6-P were converted to carbon dioxide, ribulose 5-phosphate (Ru5P) and reduced NADPH, while also replenishes glycolytic intermediates for energy production. Glycolysis also constitutes a branch of the PPP, providing glucose-6-phosphate dehydrogenase (G6PD), the first enzyme in the PPP, with its substrate G-6-P. G6PD convert G-6-P to 6-phosphogluconate (6-PG), followed by converted by 6-Phosphogluconate dehydrogenase (6PGD) to produce Ru5P. The activation of PPP promotes the generation of NADPH, a coenzyme necessary for reductive biosynthesis. The PPP is one of the key pathways for scavenging intracellular ROS, maintaining redox homeostasis and synthesizing building blocks for cell survival and proliferation. In turn, the carbon dioxide produced via the PPP contributes to the acidification of the TME. Oxidants such as ROS play a crucial role in chemoresistance in cancer cells and in acidic TME. It has been shown that activation of the PPP and increased G6PD activity are necessary for some aspects of MDR in cells, especially sustaining high content of GSH that accelerates ABC transporter proteins to discharge anticancer drugs out of the cell (Polimeni et al., 2011). Importantly, MDR cells exhibit a different metabolic profile characterized by decreased PPP flux and OXPHOS rate as well as increased glycolysis and glutathione metabolism compared with their drug-sensitive counterparts (Lopes-Rodrigues et al., 2017). Recent evidence has revealed that impaired PPP disrupts redox-cycling and results in ROS overproduction, which induces Chk2/p53/NF-κB pathway-mediated P-gp upregulation and cell cycle arrest (Wang W. et al., 2018). This study provides an association between intrinsic small molecule metabolites derived from PPP and intracellular redox status in the development of MDR. However, the connection between the regulation of redox homeostasis by the PPP and the expression of ABC transporter proteins remains controversial. In addition, ribose 5-phosphate generated by transketolase (a key enzyme in non-oxPPP) is a raw material for nucleotide biosynthesis and DNA damage response, suggesting that PPP may contributes to MDR by assisting the repair of DNA damage caused by chemotherapeutic agents (Zhen et al., 2024). Suppression of the PPP was found to increase glycolysis and decrease OXPHOS activity in drug-sensitive cells, leading to a metabolic phenotype similar to that observed in multidrug resistant cells. Because some intermediates of glycolysis and the PPP are the same, multidrug resistant cells may mobilize these intermediates primarily for the synthesis of macromolecules and the production of the energy required to complete glycolysis.

## 6 Targeting reprogramming of glucose metabolism in multidrug resistant tumors

**FIGURE 4:**
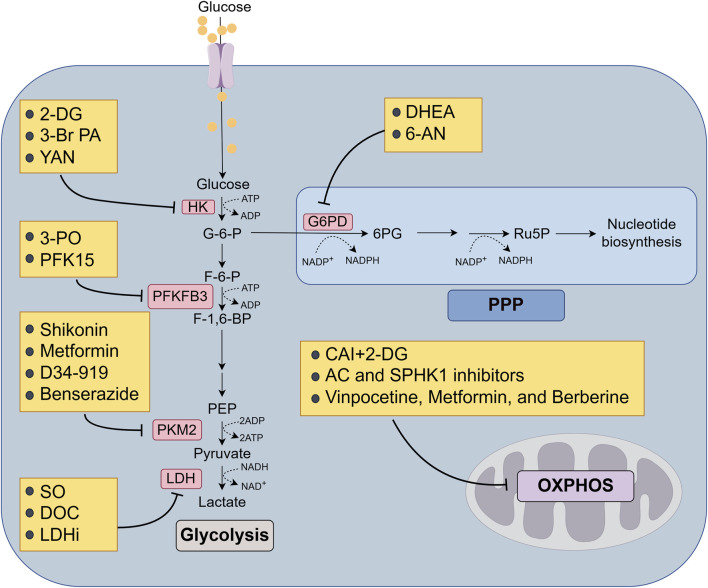
Targeting glucose metabolic enzymes and glucose metabolism to overcome MDR of cancer. Arrows indicate activation and bars mean inhibition. 2-DG, 2-deoxy-D-glucose; 3-Br PA, 3-bromopyruvate; SO, sodium oxamate; DOC, doxorubicin; LDHi, LDH inhibitors; DHEA, dehydroepiandrosterone; 6-AN, 6-aminonicotinamide; CAI, carboxyamidotriazole; AC, acid ceramidase; SPHK1, sphingosine kinase 1.

ABC transporter proteins play a pivotal role in MDR and are thus prime targets for reversing drug resistance. However, inhibition of these transport proteins was soon found to be an ineffective approach, as nonmalignant cells may also express these transport proteins, and inhibitors can cause unacceptable toxicity (Vidal et al., 2018). Given the significant role of glucose metabolism in the progression of MDR, numerous pharmacological agents are currently under development to inhibit glucose metabolism (Table 2). Targeting glucose metabolic enzymes and implementing glucose metabolism are increasingly recognized as promising strategies for addressing MDR in cancer treatment (Figure 4).

**TABLE 2:** Targeting reprogramming of glucose metabolism in multidrug resistant tumors.

Drug	Function	Treatment	References
2-DG	Glucose analogues that compete for excess glucose transporter proteins	Trastuzumab resistant breast cancer cells	Zhao et al. (2011)
3-Br PA	Induces covalent modification of HK2 protein, dissociation from mitochondria	Increases sensitivity of HCC cells to sorafenib	Yoo et al. (2019)
	Promotes autophagy-dependent ferroptosis in CRC	Overcomes both the intrinsic and acquired cetuximab resistance	Mu et al. (2023)
	Inhibition of P-gp by decreasing intracellular ATP content and HK2 activity	Doxorubicin resistant MCF-7 cells	Mu et al. (2023)
YAN	Inhibition of HK2 activation PI3K/Akt/c-Myc/HIF-1α pathway	Induction of mitochondrial apoptosis in paclitaxel resistant NSCLC cells	Gao et al. (2022)
Oxamate	LDHA inhibitor inhibits glycolytic flux and counteracts Akt/mTOR/c-Myc signaling	Adriamycin insensitive K562/MDR cells	Zhang et al. (2017)
Sodium oxamate	Competes with pyruvate for binding to LDHA.	Prostate cancer cells, paclitaxel resistant cells	Muram et al. (2019), Zhou et al. (2010)
3-PO	Inhibition of the catalytic activity of PFKFB3	Inhibition of proliferation of TAM and paclitaxel resistant breast cancer cells	Truong et al. (2021)
PFK15	Inhibition of PFKFB3 leads to G0/G1 arrest and induction of mitochondrial apoptosis	Treatment of EGFR-TKI resistant NSCLC	Lypova et al. (2019)
HKB99	Blocks conformational change of PGAM1 during catalytic process	Erlotinib resistant NSCLC	Huang et al. (2021a)
Shikonin	Inhibition of PKM2 activity	Improves the sensitization of invasive breast cancer cells to paclitaxel	Li et al. (2014)
Metformin	Reduces PKM2 expression	Reverses the MDR phenotype of osteosarcoma stem cells	Shang et al. (2017)
	Inhibition of OXPHOS	Ara-C resistant AML cells	Kao et al. (2019)
	Combination with mIDH1 enzyme inhibitors, impaired mitochondrial activity	IDH mutant AML cells	Stuani et al. (2021)
IACS-010759	Combination with mIDH1 enzyme inhibitors, impaired mitochondrial activity	IDH mutant AML cells	Stuani et al. (2021)
	Combines with LDH inhibitors to inhibit OXPHOS	Tumors undergoing metabolic rewiring after the effects of LDH inhibition	Oshima et al. (2020)
miR-122	Reduces PKM2 mRNA levels	Resensitizes 5-FU resistant colon cancer cells to 5-FU	Zheng et al. (2021)
Dehydroepiandrosterone, 6-aminonicotinamide	Reduces G6PD expression and GSH levels	Doxorubicin resistant CRC cells	Polimeni et al. (2011)
Carboxyamidotriazole	Inhibition of mitochondrial complex I	Enhances the anti-cancer effect of 2-DG	Ju et al. (2016)
VLX600	Mitochondrial inhibitors	Enhanced the anticancer effects of imatinib in imatinib-resistant cells	Vitiello et al. (2018)
Mubritinib	Inhibits NADH dehydrogenase activity	Chemotherapy resistant acute myeloid leukaemia cells	Baccelli et al. (2019)
Phenetidine	Disruption of the mitochondrial respiratory complex I	Increases the tumoricidal effect of gemcitabine	Masoud et al. (2020)

### 6.1 Inhibition of HK2

The deletion of HK2 can inhibit tumor cell proliferation, altering tumor metabolism with few side effects. Therefore, targeting HK2 is a feasible strategy for treating tumors and overcoming drug resistance. Two currently available inhibitors, namely, 2-DG and 3-bromopyruvate (3-Br PA), have been used in clinical trials. 2-DG, a synthetic glucose analog, can be used as a competitive inhibitor of glucose metabolism, inhibiting glycolysis via its actions on HK. 2-DG can cross the blood‒brain barrier and be transported into cells mainly through the glucose transport proteins GLUT1 and GLUT4. 2-DG is phosphorylated intracellularly to form 2-deoxy-D-glucose-6-phosphate (2-DG-6-P). Unlike G-6-P, 2-DG-6-P lacks a 2-hydroxy group and cannot be converted to fructose-6-phosphate; thus, it cannot be metabolized by cells, leading to the accumulation of 2-DG-6-P, with subsequent impairment of glycolysis. Zhao *et al.* reported that 2-DG can synergistically prevent the growth of trastuzumab resistant breast cancer cells when administered in combination with trastuzumab (Zhao et al., 2011). Treatment with 2-DG enhances the level of glucose transporter protein, which allows elevated uptake of 2-DG and subsequent induction of breast cancer cell death (Aft et al., 2002). YAN, a novel microtubule protein inhibitor, has been shown to repress the expression and function of P-gp and MRP1 protein and induce mitochondrial apoptosis by blocking the activation of HK2-mediated PI3K/Akt/c-Myc/HIF-1α pathway in paclitaxel resistant A549 cells. Notably, 2-DG ameliorates the anti-tumor efficiency of YAN against paclitaxel resistant cells, providing a reference for overcoming mitotic slippage-triggered drug resistance (Gao et al., 2022). 3-Br PA is as an alkylating small molecule with a structure similar to those of lactate and pyruvate. 3-Br PA enables to covalently modify to HK2 protein and directly triggers its dissociation from mitochondria, which attenuates glycolytic capacity and promotes the apoptosis and death of tumor cells due to an insufficient energy supply. To weaken the resistance of tumor cells to chemotherapy, 3-Br PA can be administered in conjunction with chemotherapeutic drugs. Yoo *et al.* reported that 3-Br PA increased the susceptibility of HCC cells to sorafenib (Yoo et al., 2019). 3-Br PA overcomes both the intrinsic and acquired cetuximab resistance by facilitating autophagy-dependent ferroptosis in CRC (Mu et al., 2023). Additionally, 3-Br PA inhibits P-gp mediated efflux through reduced intracellular ATP contents and HK2 activity in doxorubicin resistant MCF-7 cells (Mu et al., 2023). Unfortunately, there are many biochemical and practical problems in clinical application of 3-Br PA. For instance, 3-Br PA is rapidly inactivated by thiol groups, leading to the resistance in glutathione-rich tumors without targeted selectivity. 3-Br PA causes burning venous sensation during intravenous infusion and does not cross the blood-brain barrier. More importantly, 3-Br PA easily pumps out from cells due to enhanced permeability, which may not achieved enough accumulation of 3-Br PA with tumor tissue to obtain the desired cytotoxic effects (El, 2018). Thus, these obstacles should be solved for improving the anticancer activity of 3-Br PA.

### 6.2 Inhibition of PFKFB3

Several PFKFB3 inhibitors, such as 3-PO, which interferes with the catalytic activity of PFKFB3 and results in cytotoxicity, apoptosis, and growth inhibition in various types of tumor cells, have been discovered. PFKFB inhibitors in combination with ER-targeted therapy have been shown to efficiently block tumor sphere formation in a variety of advanced breast cancer models, including models of TAM and paclitaxel resistance (Truong et al., 2021). PFK15, a first-in-class small molecule antagonist of PFKFB3, blocks cell cycle proteins to induce G0/G1 arrest and induces mitochondrial apoptosis. Because PFKFB3 is upregulated in EGFR-TKI resistant NSCLC, PFK15 exhibits synergistic impact with erlotinib in wild type and EGFR mutant cell lines (Lypova et al., 2019). Strikingly, PFK15 has already shown benefit for patients with advanced solid tumors and no adverse effects on blood glucose, erythrocyte or leukocyte concentrations in a phase I clinical trial. Further research and development of new PFKFB3 inhibitors is crucial for the effective treatment of drug resistance tumors in combination with chemotherapy or targeted therapies.

### 6.3 Inhibition of PKM2

Based on the indispensable role of PKM2 in the regulation of glycolysis and cell survival, PKM2 is a potential target for overcoming drug resistance. High expression of PKM2 is associated with resistance to chemotherapeutic agents such as cisplatin and carboplatin (Wang X. et al., 2017; Liu et al., 2017). In contrast, PKM2 silencing was found to restore oxaliplatin sensitivity in oxaliplatin resistant colorectal cancer cells (Lu et al., 2017). Self-assembled hyaluronan nanoparticles encapsulating double-stranded small interfering RNA (siRNA) oligonucleotides targeting PKM2 and MDR1 increased the efficacy of paclitaxel in multidrug resistant ovarian cancer (Talekar et al., 2015). Shikonin is a small molecule naphthoquinone compound extracted from comfrey and is the most potent and specific PKM2 inhibitor reported to date. Treatment with shikonin improves the sensitization of invasive breast cancer cells to paclitaxel (Li et al., 2014). Due to its natural composition, shikonin can induce cytotoxic effects through various mechanisms. However, its complex pharmacological activity and limited solubility have hindered its clinical application (Boulos et al., 2019). Hence, future research should be focused on refining the chemical structure of L-shikonin and its derivatives, exploring appropriate drug formulations to increase its solubility, and establishing a comprehensive system for clinical drug evaluation. Metformin, a widely prescribed medication for type II diabetes, has shown to reverse the MDR phenotype of osteosarcoma stem cells by reducing PKM2 expression (Shang et al., 2017). Glycolysis is highly activated in 5-FU resistant colon cancer cells (He et al., 2014), yet miR-122 directly interacts with the 3'UTR of PKM2 to decrease PKM2 mRNA level and resensitize tumor cells to 5-FU. Zheng et al. reported that the long noncoding RNAs (lncRNAs) XIST could competitively bind to miR-137, thereby enhancing the PKM2 level and promoting chemoresistance in colon cancer cells (Zheng et al., 2021). LncRNA FEZF1-AS1 assists glycolytic ability in tumor cells by increasing the stability and expression of PKM2 (Bian et al., 2018). Moreover, lncRNA SNHG6 binds to hnRNPA1 and subsequently facilitates hnRNPA1-mediated splicing of PKM, resulting in an increased PKM2/PKM1 ratio, aerobic glycolysis and carcinogenic progression in colorectal cancer (Lan et al., 2020). Considering that many functional lncRNAs in tumor cells are involved in the acquisition of drug resistance by affecting the expression of glucose-metabolizing enzymes or activating metabolism-related pathways, focusing on the role of these lncRNAs in tumor metabolism and exploring detailed mechanisms may also identify potential targets for the early detection and treatment of tumors. The small molecule compound D34-919 can block the interaction between ALDH1A3 and PKM2, thereby reverse the tetramerization of PKM2 and restores GBMs cells sensitivity to chemoradiotherapy without affecting the metabolic function of normal cells (Li et al., 2024). Benserazide, a PKM2 inhibitor, can directly bind to PKM2 to block its activity and inhibit glycolysis, resulting in the activation of OXPHOS (Zhou et al., 2020). Therefore, it may be considered in combination with OXPHOS inhibitors to restore drug sensitivity in cancer cells.

### 6.4 Inhibition of LDHA

Sodium oxamate (SO) is a representative drug that inhibits lactate dehydrogenase and exerts its pharmacological effects by competing with pyruvate for binding to LDHA. Inhibition of LDHA activity with doxorubicin (DOC) reverses the sensitivity of prostate cancer cells (Muram et al., 2019), It also reverses the sensitivity of paclitaxel resistant cells to paclitaxel (Zhou et al., 2010). However, the inhibitory effect of SO on LDHA is non-specific, so more efficient and targeted drugs need to be explored.

### 6.5 Inhibition of OXPHOS

Metabolic plasticity allows the metabolic patterns of cancer cell subpopulations to switch between glycolysis and OXPHOS to promote cell survival during exposure to chemotherapy or targeted therapy (Guerra et al., 2017). Therefore, targeted inhibition of the glycolytic pathway may result in metabolic reprogramming to favor an OXPHOS-dependent metabolic pathway in cancer cells. There seems to be a strong link between acquired drug resistance and increased OXPHOS activity, which clarifies the ability of OXPHOS inhibitors to effectively counteract drug resistance in various tumors. Carboxyamidotriazole (CAI) is an anticancer compound that acts as a non-voltage-gated calcium channel agonist. It is speculated to inhibit tumor growth and metastasis potentially by functioning as a mitochondrial complex I inhibitor (Ju et al., 2016). When combined with 2-DG, CAI enhances the anticancer effect of 2-DG and has a dual inhibitory effect on energy production. Chemotherapy is a common effective treatment approach for B-cell acute lymphoblastic leukemia (B-ALL), but 20% of patients experience tumor recurrence despite treatment with chemotherapeutic agents such as cytarabine (Ara-C) due to a shift in metabolic flux toward OXPHOS (Chen et al., 2021). AML is another hematological malignancy with a poor prognosis. The expression and enzymatic activity of acid ceramidase (AC) and sphingosine kinase 1 (SPHK1) were found to be greater in AML cells resistant to daunorubicin and Ara-C than in the parental cells, accompanied by the upregulations of OXPHOS activity. Administration with AC and SPHK1 inhibitors partially reversed mitochondrial biogenesis and drug resistant phenotype (Kao et al., 2019). Furthermore, the effective attenuation of Ara-C induced resistance was observed both *in vivo* and *in vitro* through the inhibition of OXPHOS using three drugs, namely, vinpocetine, metformin, and berberine. Twenty percent of patients with AML acquire isocitrate dehydrogenase (IDH) mutations and exhibit increased mitochondrial metabolism, which triggers the abnormal accumulation of α-ketoglutarate and leukemogenesis (Liu and Gong, 2019). However, IDH inhibitors fail to reverse fatty acid oxidation and OXPHOS. Indeed, IDH1 inhibitors suppress Akt activity and promote mitochondrial complex I activity, resulting in the activation of PGC1α and subsequent upregulation of OXPHOS. Accordingly, blockage of OXPOHS can improve the anticancer effect of IDH mutant inhibitors (Stuani et al., 2021). It has been demonstrated that IDH mutant AML cells show enhanced vulnerability to various small molecules of OXPHOS, and the combination of ETC complex I inhibitor: metformin, IACS-010759, and ETC complex III inhibitor ATVQ in combination with an active inhibitor of isocitrate dehydrogenase 1 mutant enzyme (mIDH1 enzyme) impairs mitochondrial activity and increases anti-leukemia efficacy. Although IDH inhibitors have generally shown a favorable clinical response, the persistent challenge of primary and acquired resistance necessitates further attention. In addition, the combination of LDH inhibitors (LDHi) and IACS-010759 can minimize the targeted systemic toxicity of LDH inhibitors and significantly improve anti-tumor activity (Oshima et al., 2020).

### 6.6 Inhibition of G6PD

In doxorubicin resistant CRC cells, there was an upregulation of G6PD activity, and the overexpression of G6PD further contributed to the MDR phenotype. The application of G6PD inhibitors, specifically DHEA (dehydroepiandrosterone) and 6-AN (6-aminonicotinamide), effectively reduced G6PD expression and GSH levels, thereby inhibiting the MDR of doxorubicin resistant CRC cells (Polimeni et al., 2011). These findings suggest that targeting the PPP pathway represents a promising therapeutic strategy.

### 6.7 Inhibition of tyrosine kinases

Most gastrointestinal stromal tumors (GISTs) are characterized by the presence of activating mutations in the Kit receptor tyrosine kinase (RTK). Imatinib (STI571) can potently inhibit RTKs to delay disease progression and significantly improve the outcomes of patients with GISTs. However, a small proportion of patients exhibit intrinsic resistance to imatinib, while the majority of patients develop acquired resistance after approximately 2 years of imatinib treatment. Imatinib impedes glycolysis in cancer cells, leading to decreased glucose uptake and triggering an increase in OXPHOS activity, which is the primary driver of drug resistance. The addition of the mitochondrial inhibitor VLX600 enhanced the anticancer effects of imatinib in imatinib resistant cells (Vitiello et al., 2018). Mubritinib (an ERBB2 inhibitor) was recently shown to exert strong anticancer effects on chemotherapy resistant AML cells (Baccelli et al., 2019). Mubritinib has been demonstrated to suppress NADH dehydrogenase activity in a ubiquinone-dependent manner, thereby inhibiting ETC complex I and OXPHOS in AML cells. A phase I clinical trial of mubritinib in ERBB2+ solid tumors was successfully completed, suggesting a great potential for expedited integration of mubritinib into treatment regimens for AML. Ibrutinib is a covalent inhibitor of Bruton’s tyrosine kinase (BTK), which is a crucial player in the development of mantle cell lymphoma (MCL) and is significantly upregulated in MCL cells. Although ibrutinib has shown encouraging results in the treatment of MCL, the emergence of acquired resistance in MCL cells remains an ongoing clinical challenge, with alterations in the BCR-BTK pathway potentially contributing significantly to resistance. Studies have revealed that ibrutinib resistant MCL cells exhibit elevated expression levels of OXPHOS-related genes, suggesting that increased activation of OXPHOS pathways is the primary mechanism driving drug resistance (Fuhr et al., 2022). These findings indicate that the combination of OXPHOS inhibitors with tyrosine kinase inhibitors may be a viable strategy for cancer therapy.

### 6.8 Utilization of biguanide inhibitors

Metformin has made an indelible contribution to reducing mortality and prolonging survival in many cancers and the possible mechanism by which metformin exerts its anticancer effect may involve interference with mitochondrial ETC complex I activity and HIF-1 expression (Wheaton et al., 2014). In pancreatic cancer, mitochondria-targeted metformin (MitoMet) exhibits 1,000 times greater efficacy than metformin in inducing cell death, accompanied by ROS elevation and AMPK activation, while had no obvious cytotoxicity in non-transformed cells (Cheng et al., 2016). Phenetidine, another biguanide, also has the ability to disrupt mitochondrial respiratory complex I. Phenetidine has a higher affinity for mitochondria than metformin and is more likely to penetrate the cell membrane. This property of phenetidine may counteract the resistance observed in pancreatic cancers with high OXPHOS activity, which are resistant to conventional chemotherapeutic drugs such as gemcitabine (Masoud et al., 2020). PDAC cells are highly plastic in terms of energy metabolism, and phenetidine is more than sufficient to induce a shift in the glycolytic capacity of PDAC cells to a hypo-OXPHOS phenotype, thereby increasing the tumoricidal effect of gemcitabine. Phenetidine has been discontinued from clinical use in some countries due to its tendency to induce lactic acidosis in diabetic patients. However, by exercising increased clinical vigilance, this adverse effect can be prevented. The use of mitochondrial complex inhibitors in combination with chemotherapeutic agents or targeted agents for the treatment of drug resistance tumors is a promising strategy. However, it is important to consider the specific characteristics of different types of tumor cells when selecting mitochondrial complex inhibitors. For example, metformin supplementation does not increase the efficacy of gemcitabine or erlotinib in the management of PDAC. Phenformin selectively triggers apoptosis in Kras mutation and LKB1-deficient NSCLC cells, but not in those with Kars and p53 mutations. Loss of LKB1 results in inactivation of AMPK, which is unable to sense metabolic stress when OXPHOS is compromised (Shackelford et al., 2013). Phenformin remarkably delays the development of resistance to BRAF inhibitor PLX4720 in BRAF-mutated melanoma cells (Yuan et al., 2013). A recent study revealed that phenformin promotes cell death by inducing autophagy and apoptosis through the action of new targets, such as DDIT4 and NIBAN1, in oral squamous cell carcinoma (Zhuang et al., 2024). Anti-apoptosis and autophagy are among the main ways to maintain the malignant proliferation of MDR cells, so the therapeutic effect of phenformin in MDR cancer is worthy of further exploration.

In conclusion, biguanide inhibitors and other mitochondrial complex I inhibitors have shown considerable promise in overcoming tumor resistance to chemotherapy and targeted drugs, and the application of OXPHOS-related inhibitors and metformin analogues for cancer therapy needs to be examined in future clinical trials.

## 7 Conclusion and perspectives

Reprogramming of energy metabolism has been extensively recognized as a key hallmark of tumor cells. Aerobic glycolysis not only supports the accumulation of glycolytic intermediates required for macromolecule biosynthesis and cell division but also provides intracellular reducing capacity via the PPP, which contributes to cell survival, cell proliferation, malignant progression and drug resistance in cancers. Moreover, glycolytic fueling has been demonstrated to be associated with the activation of oncogenes and mutation of tumor suppressors, and this reliance can be increased under hypoxic conditions. Alterations in oncogenes and/or tumor suppressors in turn augment aerobic glycolysis by increasing the expression of glucose transporters and glycolytic enzymes (Hanahan and Weinberg, 2011). Therefore, the development of small molecule inhibitors of metabolic enzymes is considered a promising and beneficial strategy for improving the outcomes of cancer therapy. Numerous glucose metabolism inhibitors have shown potent anticancer effects and favorable safety profiles in preclinical studies and/or early phases of clinical trials. However, several challenges remain in their further clinical translation (Bukowski et al., 2020): Metabolic enzymes are expressed as multiple isoforms that generally cannot be distinguished by small molecule inhibitors, possibly resulting in substantial toxicity or off-target effects in normal cells (Kang et al., 2007). Cancer cells exhibit different subpopulations according to their energy production profiles. Hypoxic tumor cells principally utilize glucose as the main energy source and secrete lactate, while oxygenated tumor cells preferentially import and consume lactate for oxidative metabolism via the TCA cycle (Sonveaux et al., 2008). Importantly, the oxygen concentration, ranging from “normoxic” environment to hypoxia, temporally and regionally fluctuates within tumor tissues due to the disorganization and variability of the tumor-associated neovasculature (Hardee et al., 2009). The metabolic heterogeneity of cancer cells may result in failure of chemotherapy (Vasan et al., 2019). Alternative macromolecular metabolism pathways, including pathways of glutamine, fatty acid, and purine and pyrimidine base metabolism, exhibit compensatory upregulation to protect tumor cells against small molecule inhibitors targeting glucose metabolism, highlighting the crosstalk in the regulation of tumor metabolic plasticity. Thus, combination therapy with other metabolic inhibitors or other therapeutic modalities may exhibit great synergistic effects to improve sensitivity to chemotherapy (Boumahdi and de Sauvage, 2020). Metabolites in the TME also play critical roles in modulating drug resistance. The elevate content of lactate secreted from tumor cells facilitates the creation of an immunosuppressive niche by decreasing monocyte migration and T-cell activation as well as stimulating macrophage repolarization from the M1 to the M2 phenotype as well as angiogenesis. Reciprocally, the microenvironment alters the metabolic profile, redox homeostasis, cellular signaling responses and transcriptional programs of cancer cells to accelerate the development of tolerance to chemotherapy (Pavlova et al., 2022).

In summary, MDR in tumors is a complex and multifactorial process, and many different molecular mechanisms have been identified. Therefore, a better and deeper understanding of the genetic and epigenetic basis of the role of dysregulated glucose metabolism in modulating the chemoresistance of tumor cells will lead to the identification of novel therapeutic targets and strategies, which may be helpful for overcoming clinical treatment failure and prolonging patient survival. In addition, more attention must be devoted to the metabolic interactions between tumor cells and the surrounding microenvironment. Based on the evidence indicating that tumor cells can utilize various other nutrients, including essential fatty acids, vitamins, cysteine and methionine, studies must be conducted to investigate how these nutrients contribute to MDR.

## Glossary

MDR: multidrug resistance
TMDs: transmembrane structural domains
ABC: ATP-binding cassette
P-gp: P-glycoprotein
MRP1: multidrug resistance-associated protein 1
BCRP: breast cancer drug resistance protein
MDR1: multidrug resistance protein 1
ABCG2: Adenosine triphosphate (ATP)-binding cassette transporter G2
EV: extracellular vesicle
ALL: acute lymphoblastic leukemia
AML: acute myeloid leukemia
NSCLC: non-small cell lung cancer
PI3K: Phosphatidylinositol 3-kinase
Akt: protein kinase B
CSC: cancer stem cell
NF-κB: nuclear factor kappa-B
TME: tumor microenvironment
OXPHOS: oxidative phosphorylation
PPP: pentose phosphate pathway
NADPH: nicotinamide adenine dinucleotide phosphate
BCRP: breast cancer resistance protein
ERK1/2: extracellular signal-regulated kinase 1/2
EGFR: epidermal growth factor receptor
FGFFR: fibroblast Growth Factor Receptor
PIM2: Pim-2 proto-oncogene, serine/threonine kinase
HCC: hepatocellular carcinoma
TAM: tamoxifen
GLS/GAC-GLUL: glutaminase-glutamine synthetase
ROS: reactive oxygen species
ETC: electron transport chain
TNBC: triple negative breast cancer
HK: hexokinase
PGI: phosphohexose isomerase
PFK1: phosphofructokinase-1
TPI: triosephosphate isomerase
GAPDH: glyceraldehyde-3-phosphate dehydrogenase
PGK1: phosphoglycerate kinase 1
PGAM: phosphoglycerate mutase
LDH: Lactate dehydrogenase
G-6-P: glucose-6-phosphate
VDAC: Voltage-dependent anion channel
ER: estrogen receptor
MAPK: mitogen-activated protein kinase
F-6-P: fructose-6-phosphate
F-1,6-BP: fructose 1,6-bisphosphate
PFKP: PKF1 platelet isoform
KAT5: lysine acetyltransferase 5
PFKFB: 6-phosphofructo-2-kinase/fructose-2,6-bisphosphatase
ERCC1: external RNA Controls Consortium 1
SRC-3: steroid receptor cofactor-3
AMPD1: adenosine monophosphate deaminase 1
XDH: xanthine dehydrogenase
DHAP: dihydroxyacetone phosphate
G-3-P: glyceraldehyde 3-phosphate
OS: overall survival
LUAD: lung adenocarcinoma
1,3-BPG: 1,3-bisphosphoglycerate
PDAC: pancreatic ductal adenocarcinoma
PDHK1: pyruvate dehydrogenase kinase 1
TCA: tricarboxylic acid
O-GlcNAc: O-linked N-acetylglucosamine
CDC7: cell division cycle 7
CtIP: CTBP-interacting protein
PARP: poly ADP-ribose polymerase
2-PG: 2-phosphoenolpyruvate
PEP: phosphoenolpyruvate
MSE: muscle-specific enolase
HDAC1: histone deacetylase 1
PPIs: proton pump inhibitors
GBM: recent studies have found that glioblastoma
XRCC1: X-ray cross-complementary protein 1
ALDH: aldehyde dehydrogenase
PDH: pyruvate dehydrogenase
PDC: pyruvate dehydrogenase complex
acetyl-CoA: acetyl coenzyme A
PDK: pyruvate dehydrogenase kinase
COL11A1: collagen type XI alpha 1
DCA: dichloroacetate
HSF1: heat shock factor 1
α-KGDH: α-Ketoglutarate dehydrogenase
FH: Fumarate hydratase
PTEN: phosphatase and tensin homolog deleted on chromosome ten
2-DG: 2-deoxy-D-glucose
HGSOC: high-grade serous ovarian cancer
PGC1α: peroxisome proliferator-activated receptor gamma (PPARγ) coactivator 1 alpha
IL-6: interleukin 6
G6PD: glucose-6-phosphate dehydrogenase
3-Br PA: 3-bromopyruvate
siRNA: small interfering RNA
lncRNAs: long noncoding RNAs
SO: sodium oxamate
CAI: Carboxyamidotriazole
B⁃ALL: B⁃cell acute lymphoblastic leukemia
Ara-C: cytarabine
2-DG-6-P: 2-deoxy-D-glucose-6-phosphate
DOC: doxorubicin
AC: acid ceramidase
SPHK1: sphingosine kinase 1
IDH: isocitrate dehydrogenase
LDHi: LDH inhibitors
GISTs: Most gastrointestinal stromal tumors
RTK: receptor tyrosine kinase
BTK: Bruton’s tyrosine kinase
MCL: mantle cell lymphoma
MitoMet: mitochondria-targeted metformin
VEGF: vascular endothelial growth factor
CMTM6: CKLF Like MARVEL Transmembrane Domain Containing 6
PK: Pyruvate kinase
ATM: ataxia telangiectasia mutated
DSB: DNA double-strand break
HR: homologous recombination
Ru5P: ribulose 5-phosphate
6-PG: 6-phosphogluconate
3-PGA: 3-phosphoglycerate
2-PGA: 2-phosphoglycerate
